# Investigating the therapeutic effects and mechanisms of *Carthamus tinctorius L*.-derived nanovesicles in atherosclerosis treatment

**DOI:** 10.1186/s12964-024-01561-6

**Published:** 2024-03-12

**Authors:** Rongfeng Yang, Fengxia Lin, Wenlin Wang, Gang Dai, Xiao Ke, Guifu Wu

**Affiliations:** 1 Department of Cardiology, The Eighth Affiliated Hospital, Sun Yat-sen University, Shenzhen, China; 2 Guangdong Innovative Engineering and Technology Research Center for Assisted Circulation, Shenzhen, China; 3 NHC Key Laboratory of Assisted Circulation (Sun Yat-sen University), Shenzhen, China; 4 Department of Cardiology, Fuwai Hospital, Chinese Academy of Medical Sciences (Shenzhen Sun Yat-sen Cardiovascular Hospital), Shenzhen, China; 5 Department of Cardiology, Shenzhen Bao’an Chinese Medicine Hospital, Guangzhou University of Chinese Medicine, Shenzhen, China; 6 Department of Clinical Medicine, University of South China, Hengyang, China

**Keywords:** Nanovesicles, Atherosclerosis, miRNA, Drug delivery, Herbal medicine, Endothelial cell

## Abstract

**Background:**

*Carthamus tinctorius L.*, a traditional herbal medicine used for atherosclerosis (AS), lacks a clear understanding of its therapeutic mechanisms. This study aimed to investigate the therapeutic effects and mechanisms of *Carthamus tinctorius L.-*derived nanovesicles (CDNVs) in AS treatment.

**Methods:**

CDNVs were isolated and characterized using improved isolation methods. Transmission electron microscopy, nanoparticle tracking analysis, and protein analysis confirmed their morphology, size, and protein composition. Small RNA sequencing was performed to identify the miRNA profile of CDNVs, and bioinformatics analysis was used to determine their potential biological roles. In vivo biodistribution and toxicity studies were conducted in mice to assess the stability and safety of orally administered CDNVs. The anti-atherosclerotic effects of CDNVs were evaluated in ApoE-/- mice through plaque burden analysis. The protective effects of CDNVs on ox-LDL-treated endothelial cells were assessed through proliferation, apoptosis, reactive oxygen species activation, and monocyte adhesion assays. miRNA and mRNA sequencing of CDNV-treated endothelial cells were performed to explore their regulatory effects and potential target genes.

**Results:**

CDNVs were successfully isolated and purified from *Carthamus tinctorius L.* tissue lysates. They exhibited a saucer-shaped or cup-shaped morphology, with an average particle size of 142.6 ± 0.7 nm, and expressed EV markers CD63 and TSG101. CDNVs contained proteins, small RNAs, and metabolites, including the therapeutic compound HSYA. Small RNA sequencing identified 95 miRNAs, with 10 common miRNAs accounting for 72.63% of the total miRNAs. These miRNAs targeted genes involved in cell adhesion, apoptosis, and cell proliferation, suggesting their relevance in cardiovascular disease. Orally administered CDNVs were stable in the gastrointestinal tract, absorbed into the bloodstream, and accumulated in the liver, lungs, heart, and aorta. They significantly reduced the burden of atherosclerotic plaques in ApoE-/- mice and exhibited superior effects compared to HSYA. In vitro studies demonstrated that CDNVs were taken up by HUVECs, promoted proliferation, attenuated ox-LDL-induced apoptosis and ROS activation, and reduced monocyte adhesion. CDNV treatment resulted in significant changes in miRNA and mRNA expression profiles of HUVECs, with enrichment in inflammation-related genes. CXCL12 was identified as a potential direct target of miR166a-3p.

**Conclusion:**

CDNVs isolated from *Carthamus tinctorius L*. tissue lysates represent a promising oral therapeutic option for cardiovascular diseases. The delivery of miRNAs by CDNVs regulates inflammation-related genes, including CXCL12, in HUVECs, suggesting their potential role in modulating endothelial inflammation. These findings provide valuable insights into the therapeutic potential of CDNVs and their miRNAs in cardiovascular disease.

**Supplementary Information:**

The online version contains supplementary material available at 10.1186/s12964-024-01561-6.

## Background

Atherosclerosis (AS) is a chronic inflammatory disease that contributes to various cardiovascular disorders and imposes significant medical and economic burdens [1, 2]. Despite progress in understanding AS mechanisms and therapeutic drug development, the complete pathogenesis of AS is still not fully understood, and some patients continue to experience vascular complications [3, 4]. Therefore, the identification and development of new intervention targets for AS are active areas of research, involving the identification of new molecular mechanisms and pathways involved in AS development and evaluating the safety and efficacy of potential drugs targeting these mechanisms.

Extracellular vesicles (EVs) are nano-sized membrane-derived vesicles that are released from cells into the extracellular environment, which play an important role in cell-to-cell communication by transferring their contents such as proteins, lipids and nucleic acids between cells [5]. In recent years, there have been widespread reports on the potential therapeutic benefits of mammalian EVs for preventing AS development. EVs derived from endothelial progenitor cells, bone marrow macrophages, and thrombin-activated platelets are emerging as promising vehicles for delivering specific microRNAs (miRNAs) to treat atherosclerosis [6–8]. These studies demonstrate the potential of EV-based therapies as effective treatments for cardiovascular disease, distinguishing them from traditional drugs. However, despite their immense therapeutic potential, the clinical translation of EVs in AS is hindered by challenges related to their origin, production scalability, acquisition complexity, and ethical considerations [9].

Plant-derived nanovesicles (PDNVs) exhibit similarities to mammalian EVs in terms of their phospholipid bilayer structure and complex composition, including lipids, nucleic acids, and proteins [10]. However, PDNVs offer several advantages over mammalian EVs, making them an appealing alternative. These advantages include straightforward extraction, cost-effectiveness, high yield, potential for oral administration, and absence of zoonotic pathogens [11, 12]. Additionally, PDNVs possess various therapeutic properties, such as anti-inflammatory and antioxidant effects, as well as potential benefits for improving insulin resistance and obesity [13–15]. These properties suggest that PDNVs are a promising therapeutic option for AS treatment. However, direct evidence of the effects of PDNVs on AS is currently lacking. Thus, further research is required to elucidate the therapeutic potential of PDNVs in AS and optimize their production and purification.

*Carthamus tinctorius L.* (CTL), commonly known as safflower, is a traditional herbal medicine with well-established cardiovascular protective effects widely used in Asian countries [16]. CTL is frequently recommended for the treatment of AS-related conditions, such as coronary heart disease and ischemic stroke [17]. Although hydroxysafflor yellow A (HSYA) was previously considered the primary active compound in CTLs, its low oral bioavailability cannot fully explain the observed therapeutic effects of CTLs following oral administration [18]. Therefore, the specific mechanisms and targets of CTLs in AS treatment remain elusive.

In this study, we successfully isolated and purified nanovesicles derived from *Carthamus tinctorius L.* (CDNVs). We demonstrate that CDNVs possess potent anti-atherosclerotic effects and effectively alleviate inflammation in endothelial cells. Additionally, we identified the delivery of miR166a-3p by CDNVs as a potential mechanism by which CTLs treat AS.

## Materials and methods

### Animal ethics

The animal protocol was reviewed and approved by the Institutional Animal Care and Use Committee of Sun Yat-sen University, China (No. SYSU-IACUC-2022-000564).

### Isolation and characterization of CDNVs

To isolate and purify the CDNVs, dried CTL flowers were washed with distilled water to remove impurities. The flowers were soaked overnight in phosphate-buffered saline (PBS) and were transferred to a homogenizer (Joyoung, Y921). They were blended at the highest speed (35,000 rpm) for 1 min, followed by a 1-min pause, and this process was repeated 5 times. The resulting mixture was passed through a 20-mesh stainless-steel cell strainer to remove debris, and the filtrate was collected. The filtrate was sequentially centrifuged at 300×g, 2,000×g, and 10,000×g (JA 25.50, model: AvantiJXN-26, Beckman Coulter), and the resulting precipitate was discarded. The remaining supernatant was ultracentrifuged at 135,000×g for 70 min (SW41Ti, model: OptimaL-100XP, Beckman Coulter), and the resulting precipitate was resuspended in PBS to obtain CDNVs. A sucrose density gradient was prepared (to prepare 8%, 20%, 30%, and 50% sucrose solutions using a PBS solution, first, add 1 ml of 50% sucrose solution to the bottom of a high-speed centrifuge tube (Beckman, 331372). Then, gently and evenly add the following sucrose solutions from bottom to top: 2 ml of 30% sucrose solution, 2 ml of 20% sucrose solution, and 3 ml of 8% sucrose solution. This will create a well-defined sucrose gradient solution with distinct layers), and 2 ml resuspended CDNVs were layered on top of the gradient and centrifuged at 150,000×g for 2 h. The fraction between the 20% and 30% gradients was collected as the CDNV suspension (Additional file 1: Fig. S1A). Finally, the suspension was filtered using a 0.22 μm-filter and stored at − 80 °C.

For transmission electron microscopy analysis of the CDNVs, a droplet of the CDNV sample was deposited onto a carbon-coated copper grid with a hydrophilic surface. For negative staining, a droplet of 2% uranyl acetate solution was applied to the grid for 1 min. The samples were then analysed under a Tecnai G2 Spirit Twin Transmission Electron Microscope, and the images were recorded with a GATAN 832.10 W camera. The size distribution of CDNVs was analysed by nanoparticle tracking analysis using a Nanosight300 (Malvern Instruments, UK) with a camera level set at 12, and a total of 1643 valid tracks recorded. The zeta potential of the CDNVs was analysed using a Multiple-Laser ZetaView® f-NTA Nanoparticle Tracking Analyser (Particle Metrix, Germany). Coomassie brilliant blue staining was performed to identify proteins. We used exosome markers, including HSP-70 (Huabio, ER50802), CD63 (Huabio, ER1905-64), and TSG101 (Huabio, ET1701-59), and determined the protein concentration of the CDNVs using a BCA commercial kit (Biosharp, BL521A) according to the manufacturer’s instructions. RNA was identified by electrophoresis using a 1.2% polyacrylamide gel, and its concentration was determined using an Agilent 2100 Bioanalyzer.

### Targeted metabolomics assay

To quantify HSYA in the CDNVs, a targeted metabolomic assay was performed using ultra-performance liquid chromatography-tandem mass spectrometry (UHPLC‒MS/MS). Briefly, samples were prepared by transferring a 10-µL aliquot of each individual sample to an EP tube and adding 990 µL of methanol. After vortexing and incubation at − 20 °C, the samples were centrifuged, and a 50-µL aliquot of the clear supernatant was transferred to an auto-sampler vial for LC‒MS/MS analysis. Stock solutions were prepared by dissolving or diluting each standard (HSYA) to a final concentration of 1 mM. A mixed working standard solution was produced by transferring a 100-µL aliquot of each of the stock solutions to a 10-mL flask; a series of calibration standard solutions were then prepared by stepwise dilution of this mixed standard solution. UHPLC separation was performed using an Agilent 1290 Infinity II series UHPLC System, and an Agilent 6495 triple quadrupole mass spectrometer was used for assay development. The MRM parameters for each target analyte were optimized, and calibration curves were generated using the least-squares method with 1/x weighting. The limits of detection and limits of quantitation were determined using signal-to-noise ratios, according to the US FDA guidelines for bioanalytical method validation.

### Untargeted metabolomics assay

Untargeted metabolomics assays were performed using UHPLC‒MS. In brief, sample preparation involved mixing 30 µL of the sample with 90 µL of methanol, vortexing for 30 s, and incubating at − 20 °C for 1 h to precipitate proteins. After centrifugation at 12,000 rpm for 15 min at 4 °C, the supernatant was transferred to a fresh glass vial for LC‒MS/MS analysis. The UHPLC system (Vanquish, Thermo Fisher Scientific) was coupled to a Q Exactive mass spectrometer (Orbitrap MS, Thermo) for LC‒MS/MS analyses. The mobile phase comprised a mixture of 0.1% formic acid, 0.1% ammonia, and acetonitrile. Two columns were used sequentially: a Waters ACQUITY UPLC BEH C18 column followed by a UPLC BEH Amide column. The mobile phase flowed at 300 µL/min, and the injection volume was 2 µL. The ESI source conditions were set as follows: spray voltage = + 3,800/−3,200 V, sheath gas (N2) flow rate = 40, aux gas (N2) flow rate = 15, sweep gas (N2) flow rate = 0, aux gas (N2) temperature = 320 °C, capillary temperature = 320 °C. The acquisition parameters of the mass spectrometer were as follows: scan range: 50–750, full mass resolution: 70,000, ddMS2 resolution: 17,500, collision energy (CE): 30, and loop count: 5. LC‒MS/MS data were processed using the appropriate software to generate peak lists and perform database searches.

### CDNV labelling

For DiR labelling, CDNVs (100 mg in 1 mL PBS) were mixed with 1 mL DiR dye (Macklin, D909616, 1 mM in DMSO) and incubated at 37 °C for 30 min. For DiL labelling, CDNVs (100 mg in 1 mL PBS) were mixed with 1 mL DiL dye (Macklin, D82309, 1 mM in DMSO) and incubated at 37 °C for 30 min. For DiO labelling, CDNVs (100 mg in 1 mL PBS) were mixed with 1 mL DiO dye (zeta life, DiO-10, 1 mM in DMSO) and incubated at 37 °C for 30 min. All excess, unreacted dyes were removed by centrifugation at 135,000×g for 70 min, and the labelled CDNV pellets were resuspended in PBS.

### In vitro digestion of CDNVs

The in vitro digestion conditions were based on those of a previous study [19]. For the gastric fluid, 16.4 mL of diluted hydrochloric acid was added to approximately 800 mL of distilled water, and 10 g of pepsin was added and mixed thoroughly. The solution was then diluted to 1,000 mL with distilled water, and the pH was adjusted to 2.0 using diluted hydrochloric acid, if necessary. For the intestinal fluid, 6.8 g of potassium dihydrogen phosphate was dissolved in 500 mL of distilled water, and the pH was adjusted to 6.8 using 0.1 mol/L sodium hydroxide solution. Next, 10 g of pancreatin was dissolved in a suitable amount of water, mixed with phosphate buffer, and diluted to 1,000 mL with water. Gastric digestion simulation was performed by mixing 100 mg of CDNVs with 100 mL of gastric fluid at 37 °C for 60 min on a shaker, followed by purification through ultracentrifugation. Intestinal digestion was simulated by resuspending CDNVs digested with gastric fluid in the intestinal fluid and incubating for 60 min on a shaker. The stability of the CDNVs was evaluated by measuring the particle size and distribution using Nanosight300 before and after in vitro digestion.

### Biodistribution of orally administered CDNVs

Eight-week-old male C57BL/6 mice were purchased from Gempharmatech (Nanjing, China). Mice were divided into groups and administered CDNVs daily at a dose of 40 mg/kg. The treatment lasted for one week, and PBS and DiR dye were used as controls. The mice were euthanized 6 h after the final gavage, and blood, liver, lung, kidney, spleen, heart, and aorta tissues were collected to monitor the distribution of CDNVs using ex vivo imaging analysis. The in vivo distribution of CDNVs was evaluated by analysing, measuring, and quantifying the DiR fluorescence signal using the Aniview100 in vivo imaging system (Biolight, Guangzhou, China).

### Mouse atherosclerosis model

For the AS mouse model, 8-week-old male ApoE-/- mice were fed a high-fat diet for 12 weeks. ApoE-/- mice were purchased from Gempharmatech (Nanjing, China). CDNVs were administered by gavage or intravenous injection at a dose of 40 mg/kg, with HSYA used as a positive control at a dose of 0.27 mg/kg and PBS used as a negative control. The treatments were administered three times per week for 12 weeks. At the end of the 12-week treatment period, the mice were euthanized, and the serum, internal organs, and aortas were collected for further examination.

To investigate the effects of miR166a-3p derived from CDNVs on ApoE-/- mice fed a high-fat diet, we constructed in vivo miRNA mimics (agomiR166a-3p) and inhibitors (antagomiR166a-3p) and evaluated their effects on AS in ApoE-/- mice. agomiRNA NC and antagomiRNA NC were used as the negative control mimics and inhibitors, respectively. Mice were divided into four groups: PBS ig.+agomiRNA NC iv., PBS ig.+agomiR166a-3p iv., CDNVs ig.+antagomiRNA NC IV, and CDNVs ig.+antagomiR166a-3p iv. In the first two groups, mice were orally administered 200 µL of PBS and injected with 5 nmol of agomiRNA NC or agomiR166a-3p via tail vein injection. For the latter two groups, mice were orally administered CDNVs at a dose of 40 mg/kg and injected with 5 nmol of antagomiRNA NC or antagomiR166a-3p via tail vein injection. Each treatment was administered three times per week.

### Toxicity of CDNVs in vivo

For histopathology, H&E staining was performed on paraffin-embedded sections of internal organs. Serum was collected and rapidly frozen at − 80 °C until subsequent analysis. Serum alanine aminotransferase (ALT) and aspartate aminotransferase (AST) levels were measured to test for hepatotoxicity. The creatinine (CR) level was used to evaluate kidney injury.

### Serum lipid profiles

Serum triglyceride (TG), total cholesterol (TC), and low-density lipoprotein cholesterol (LDL-C) levels were determined using a microplate reader (Synergy H1, BIOTEK, USA) according to the manufacturers’ instructions (NJJCBIO).

### Enzyme-linked immunosorbent assay

Enzyme-linked immunosorbent assay (ELISA) was used to detect the serum levels of IL‐1β, IL-6, and chemokine ligand 12 (CXCL12) in ApoE-/- mice. Blood was harvested by removing the eyeball. After centrifugation at 3,000 rpm/min for 10 min, 10 µl of serum was used for ELISA analysis in accordance with the manufacturer’s protocol (Mlbio, Shanghai, China).

### Plaque lesion analysis

Histological analysis of the aorta trees and aortic roots was conducted as follows: both samples were fixed in a fixative solution, washed with PBS, and carefully dissected to remove excess tissue. The aorta trees were stained with Oil Red O solution for 60 min at 37 °C and differentiated in 60% isopropanol, whereas the aortic roots were cryosectioned and stained with Oil Red O and hematoxylin. In both cases, images were captured with appropriate camera settings, and the plaque area ratio was calculated using ImageJ software.

### Human umbilical vein endothelial cell treatment, transfection, and adenovirus infection

Human umbilical vein endothelial cells (HUVECs) were purchased from ZQXZBIO (Shanghai, China) and cultured in endothelial cell medium (Sciencell, 1001) comprising 500 ml of basal culture medium, 25 ml of foetal bovine serum, 5 ml of endothelial cell growth supplements, and 5 ml of penicillin/streptomycin solution at 37 °C with 5% CO_2_. Cells in the logarithmic growth phase were used for subsequent experiments. For all experiments, HUVECs were used at passages 3–7.

To transfect miRNA mimics, miRNA inhibitors, and siRNAs (RiboBio, Guangzhou, China), HUVECs were transfected with Lipofectamine 2000 (Invitrogen™, 11668027) in 12-well plates at approximately 60–70% confluence. The transfection mixture was added dropwise to the cells, which were then incubated for 1 h before the medium was replaced with complete culture medium. The cells were harvested 48 h after transfection.

Adenovirus packaging and purification were performed using an ADMAX system. Plasmids were transfected into 293A cells, and the viruses were collected after seven days, freeze-thawed, and purified using a virus concentration column. Finally, HUVECs were infected with adenovirus in 12-well plates and harvested after 48 h.

### Uptake of CDNVs by HUVECs

To evaluate the uptake of CDNVs by HUVECs, the cells were treated with DiL-CDNVs, and images of the labelled cells were acquired using laser scanning confocal microscopy. All experiments were performed in triplicate. Flow cytometry experiments were performed to evaluate the uptake efficiency of CDNVs. HUVECs were seeded in six-well plates and incubated with DiO-CDNVs for various time intervals (0, 1, 3, 6, and 12 h). After incubation, the cells were harvested and analysed using flow cytometry to determine the percentage of DiO-positive cells. DiO fluorescence was detected in the FITC channel, and the proportion of positive cells was calculated using FlowJo software. All experiments were performed in triplicate.

### Cell proliferation

The effect of CDNVs on HUVEC proliferation was investigated using the CCK-8 assay. All experiments were performed in a 96-well plate with 5,000 cells per well. The absorbance was measured at 450 nm using a microplate reader after incubation with CCK-8 reagent. All experiments were performed in triplicate.

### Cell apoptosis

The HUVEC apoptosis rate was evaluated using flow cytometry following treatment with CDNVs or HSYA in the presence of oxidized low-density lipoprotein (ox-LDL). Cells were collected by combining the supernatant and digested cells, washed with pre-cooled PBS, and divided into groups for staining with Annexin V and/or PI probes. The stained cells were detected using a flow cytometer, and the apoptosis rate was analysed using established methods and calculated using the following formula: apoptosis rate (%) = (number of cells stained with Annexin V only + number of cells stained with both Annexin V and PI)/total number of cells × 100. All experiments were performed in triplicate.

### Intracellular ROS levels

Serum-free endothelial cell medium containing the DCFH-DA probe was prepared at a dilution of 1:2,000, and the cells were incubated in the medium for 30 min. After washing with PBS, trypsin was added to each well for digestion, and complete DMEM was added to stop digestion. Cells were collected, washed, and resuspended in PBS. The cells were then detected using a flow cytometer, and threshold settings and voltage adjustments were performed during detection. The positive cell rate was calculated based on the positive group using software to fit the graph. All experiments were performed in triplicate.

### Adhesion assays

An adhesion assay was performed to evaluate the adhesion ability of HUVECs treated with CDNVs or HSYA in the presence of ox-LDL. Briefly, HUVECs were seeded in a 12-well plate. THP-1 cells were resuspended in PBS and counted, and 1 × 10^5^ cells were added to each well. After incubation with calcein AM, the cells were washed, resuspended in endothelial cell medium, and then added to the HUVEC monolayer. After 30 min of incubation, the cells were washed with PBS and viewed under an inverted fluorescence microscope. The number of green fluorescent-labelled cells was calculated using ImageJ software. Appropriate controls were included in each experiment to ensure the accuracy of the results. All experiments were performed in triplicate.

### Western blot analysis

Cells were lysed in RIPA buffer (Biosharp, Hefei, China) containing protease inhibitors. Protein concentrations were determined using a commercial bicinchoninic acid kit (Biosharp, Hefei, China). Equal amounts of protein from each sample were separated by SDS-PAGE and transferred onto PVDF membranes. After blocking with 5% skim milk in TBST and washing with TBST, the cells were incubated with primary antibodies against ICAM-1 (1:2,000, 15364-1-AP, Proteintech), VCAM-1 (1:1,000, R1512-13, Huabio), CXCL12 (1:500, 3530, CST), and GAPDH (1:10,000, 60004-1-Ig, Proteintech) at 4 °C overnight and then incubated with a secondary antibody. Protein bands were visualized using ECL substrate.

### RNA isolation and quantitative real-time PCR

To extract RNA from HUVECs, aortic tissues and CDNVs were isolated and homogenized in TRIzol reagent (Life Technologies, USA). RNA was reverse transcribed to cDNA using the Evo M-MLV Mix Kit (AG11728, ACCURATE BIOTECHNOLOGY (HUNAN) Co., LTD, Changsha, China). Quantitative real-time PCR (qPCR) analysis was performed using a LightCycler480 (Roche, USA). The primer sequences are listed in Additional file 1: Table S1. The expression of miRNAs in tissues and HUVECs were normalized to U6 snRNA, and the levels of VCAM-1, ICAM-1, and CXCL12 were normalized to GAPDH with SYBR® Green Pro Taq HS (AG11702, Accurate Biotechnology (Hunan) CO., LTD, ChangSha, China). For miRNAs in CDNVs detection, the synthetic miRNA *Caenorhabditis elegans* miR-39 (cel-miR-39; 10 fmol/µL; Sequence: 5ʹ-UCACCGGGUGUAAAUCAGCUUG-3′; AG, Accurate Biotechnology (Hunan) CO., LTD, ChangSha, China) was added to the isolated RNAs and was used as an exogenous control. The expression levels of the tested genes were determined and calculated by 2−∆∆Ct. Each data point contained at least three biological duplicates and is represented as the mean ± standard deviation.

### RNA sequencing analysis and bioinformatics

We employed miRNA and mRNA sequencing to investigate the effects of CDNVs on miRNA and mRNA expression in HUVECs. For miRNA sequencing, we constructed miRNA-seq libraries and performed sequencing to identify mature miRNAs and analyse their expression differences. For mRNA sequencing, we extracted total RNA from cells treated with or without CDNVs, constructed a poly A-enriched mRNA library, and obtained gene expression profiles.

miRanda software was used to predict the target genes of miRNAs in CDNVs. We performed dynamic programming alignment on the sequences of the 3’ untranslated (UTR) regions of mRNAs and miRNAs with the highest abundance in CDNVs, which included miR159a, miR170-5p, and miR166a-3p. Matching criteria were applied to the miRNA–mRNA pairs with a score cut off of > 135 and a total energy cut off of < − 20 kCal/mol, indicating complementary pairing and thermodynamic stability. The predicted results were filtered to remove redundant miRNA–mRNA pairs. To construct the high-confidence protein–protein interaction (PPI) network, we used the STRING database with a high-confidence filter. PPI network data were then imported into Cytoscape to construct the target PPI network. We conducted enrichment analysis of the target proteins in the DAVID database for GO terms and in the KOBAS database for KEGG pathways and diseases, with a threshold of *p* < 0.05. The results were visualized using GraphPad Prism 9.0 and the “ggplot2” package in R software.

### Dual-luciferase reporter gene assay

To verify whether CXCL12 was the direct target gene of miR166a-3p, we cloned a partial normal 3’ UTR sequence of CXCL12 and a sequence with random mutations in the miR166a-3p binding site into luciferase reporter gene plasmids to obtain CXCL12-wild type and CXCL12-mutant type plasmids, respectively. The constructed plasmids were then co-transfected with NC and miR166a-3p mimics into HEK-293T cells. The cells were collected in lysis buffer after 48 h. Luciferase activity was measured using a Dual-Luciferase Reporter Gene Assay Kit (Promega, E1910).

### Statistical analysis

Experimental data were expressed as the mean ± standard deviation and analysed using GraphPad Prism software (version 9.0). Statistical analyses were conducted using independent sample t tests for comparisons between two groups and one-way ANOVA for comparisons among three or more groups. A p value of less than 0.05 was considered statistically significant and indicated as “*” in the bar graphs. The level of significance was further denoted as “**” for *p* < 0.01, “***” for *p* < 0.001, and “ns” for non-significant results with *p* > 0.05.

## Results

### Isolation and characterization of CDNVs

Using an improved method for isolating extracellular vesicles (EVs) from mammalian cells [20], we successfully isolated and purified nanovesicles from CTL tissue lysates, which we refer to as CDNVs [21]. Each batch of CDNVs extraction initially used 100 g of dried saffron petals. The isolation process involved differential centrifugation to remove large particles, viscous proteins, and fibers, followed by ultracentrifugation to collect the CDNVs. A sucrose gradient ultracentrifugation step was used for further purification, resulting in a purified CDNV layer at the 20–30% sucrose interface (Additional file 1: Fig. S1A).

**Fig. 1:**
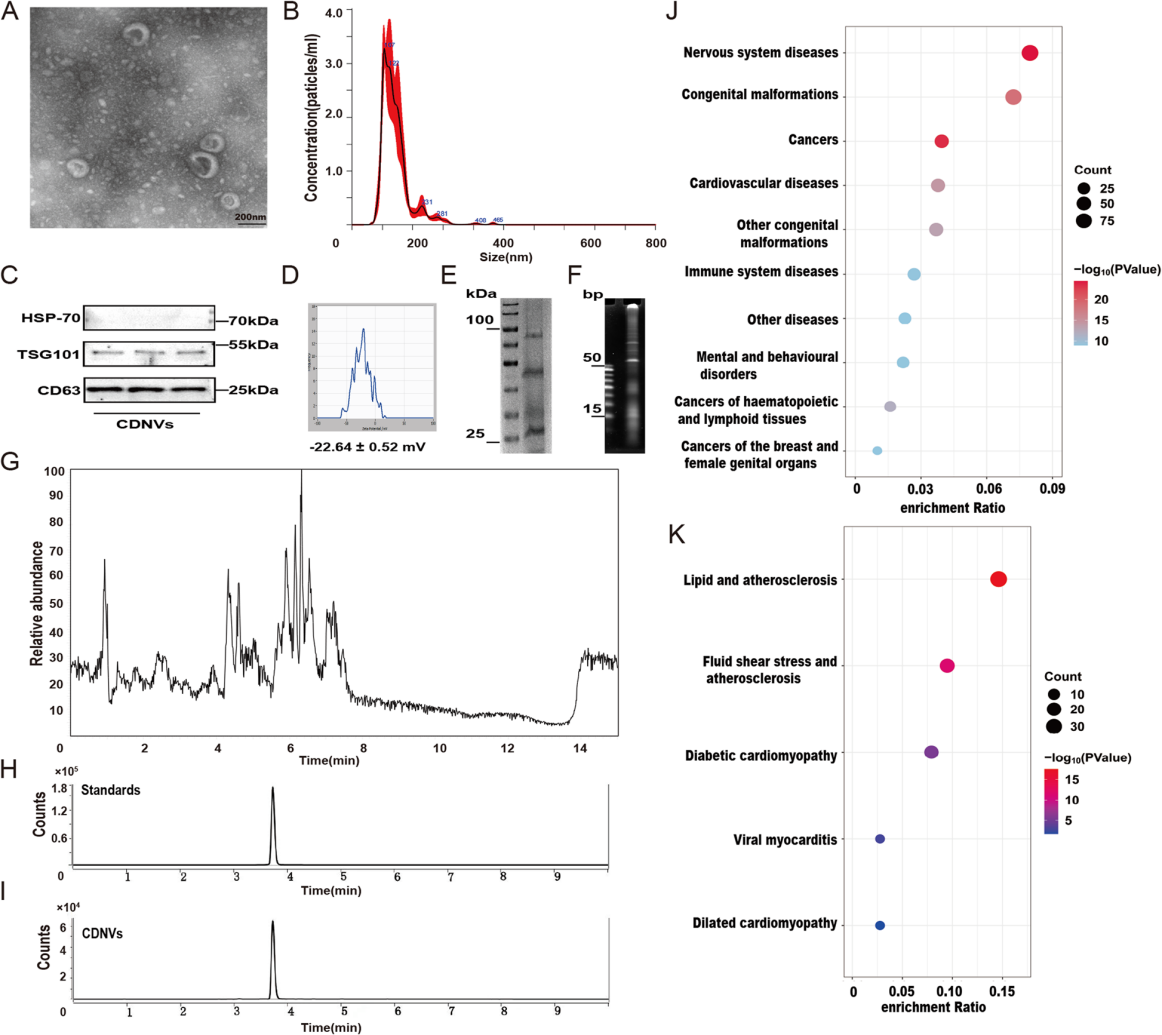
Comprehensive analysis of CDNVs: contents, miRNA profiling, and identification. **A** A representative transmission electron microscopy (TEM) image of purified CDNVs, with a scale bar representing 200 nm. **B** The average size of CDNVs was characterized by NanoSight analysis. **C** Immunoblotting images of exosomal markers in CDNVs were obtained. The three bands representing protein samples extracted from three batches of CDNVs. **D** The surface zeta potential of CDNVs was determined. **E** The proteins extracted from CDNVs were separated using SDS‒PAGE and stained using Coomassie blue dye. **F** The RNAs extracted from CDNVs were identified by electrophoresis using a 1.2% polyacrylamide gel. **G** UHPLC‒MS analysis of metabolomics extracted from CDNVs was conducted. **H** UHPLC‒MS/MS analysis of chemical reference standards of HSYA was performed. **I** UHPLC‒MS/MS analysis of HSYA extracted from CDNVs was conducted. **J** KEGG disease analysis of target genes of the top three miRNAs in CDNVs was performed. **K** KEGG pathway analysis of target genes of the top three miRNAs in CDNVs was conducted

The morphology of CDNVs was observed using transmission electron microscopy, revealing a saucer-shaped or cup-shaped structure with a characteristic lipid bilayer (Fig. 1A). Nanoparticle tracking analysis was performed to determine the size distribution of CDNVs, showing an average particle size of 142.6 ± 0.7 nm and a relatively uniform size distribution (Fig. 1B). The particle concentration of CDNVs was (11.49 ± 6.84)* 10^9^ particles per gram. Previous studies have suggested that PDNVs express mammalian EV markers, such as CD63 and TSG101 [22, 23]. Our results confirmed the presence of CD63 and TSG101 in CDNVs, indicating a degree of homology with mammalian EVs in terms of their protein composition (Fig. 1C). Zeta potential analysis showed that CDNVs have a negative charge and a relatively uniform distribution, with a single peak and an average value of − 22.64 ± 0.52 mV (Fig. 1D).

We further investigated the components of CDNVs using various analytical techniques. Coomassie brilliant blue staining revealed the presence of proteins in CDNVs, with the molecular size of the major proteins ranging from 25 kDa to 100 kDa (Fig. 1E). The amounts of proteins and nucleic acids in the CDNVs were quantified to be 39.17 ± 1.018 mg/10^11^ particles and 120.2 ± 6.702 µg/10^11^ particles, respectively (Additional file 1: Fig. S1B–C). CDNVs primarily contained small RNAs (Fig. 1F), and untargeted metabolomic analyses identified various metabolites, including arachidonic acid and luteolin (Fig. 1G). Targeted metabolomic analysis confirmed the presence of HSYA in CDNVs at a concentration of 264.2 ± 12.28 µg/10^11^ particles (Fig. 1H–I, Additional file 1: Fig. S1D). Based on these measurements, the dosing equivalent was approximately 40 mg of CDNVs (protein content contained within CDNVs) containing 0.27 mg of HSYA. These findings provide a comprehensive characterization of CDNVs and confirm their enrichment in proteins, RNAs, and metabolites.

### Small RNA sequencing of CDNVs

Small RNA sequencing was performed on three separate batches of CDNVs. Among the 95 miRNAs identified in miRBase 22.0, ten were common to all three samples, accounting for 72.63% of the total miRNAs (Additional file 1: Fig. S2A). According to the qPCR assay, the three most highly expressed miRNAs were miR166a-3p, miR159a, and miR170-5p (Additional file 1: Fig. S2B). The top three miRNAs targeted 3,702 genes. The protein-protein interaction (PPI) network of these target genes consisted of 1,191 nodes and 2,646 edges (Additional file 1: Fig. S2C). Gene Ontology (GO) enrichment analysis of these target genes in the PPI network showed that they were primarily involved in the following cellular components: nucleoplasm, cytosol; the following molecular functions: protein binding and ubiquitin protein ligase binding; and the following biological processes: cell adhesion, apoptosis, and cell proliferation (Additional file 1: Fig. S2D). Kyoto Encyclopedia of Genes and Genomes (KEGG) disease enrichment and pathway analysis of the target genes in the PPI network revealed that the miRNAs in CDNVs could affect the progression of cardiovascular diseases, with the top two pathways being lipid and AS and shear stress and AS (Fig. 1J-K). These findings provide valuable insights into the potential biological roles of miRNAs in CDNVs and their relevance in cardiovascular disease.

### In vivo biodistribution and toxicity of orally administered CDNVs

To evaluate the stability of CDNVs in the gastrointestinal tract, we simulated the conditions of gastrointestinal digestion in vitro by resuspending CDNVs in simulated gastric and intestinal fluid. Our results showed that CDNVs exhibit a certain stability in the gastrointestinal tract, which allows them to be absorbed through the gastrointestinal tract (Additional file 1: Fig. S3A-D).

**Fig. 2:**
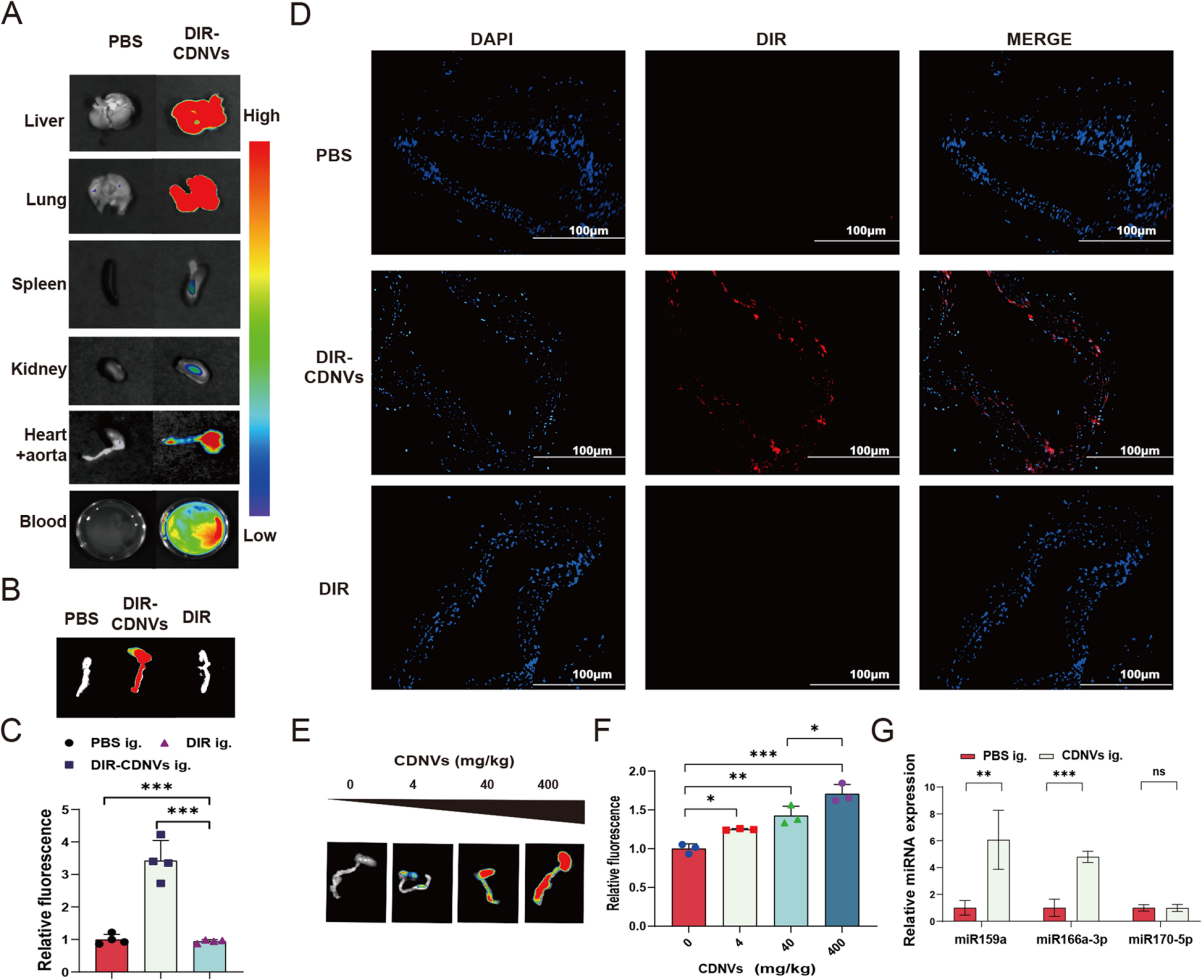
In vivo distribution of orally administered CDNVs. **A** Representative ex vivo images of various tissues, including the liver, lung, spleen, kidney, heart and aorta, were acquired using the Aniview100 In Vivo Imaging System (n = three mice per group). **B** Representative ex vivo images of the heart and aorta were obtained for the DIR-CDNVs ig. group, with DiR-free dye and PBS used as controls. **C** The fluorescence signal of DIR of the heart and aorta was quantified and statistically analysed (n = four mice per group). **D** Frozen sections of aorta were stained with DAPI, a DNA-specific dye used for cell nucleus counting and identification (blue), and the fluorescence signal of DIR (red) was observed by fluorescence microscopy (n = three mice per group). Scale bar represents 100 μm. **E** Dose-dependent fluorescence signal intensity of heart and aorta from mice after receiving gavage of DiR-CDNVs. **F** The fluorescence signal of DIR in the heart and aorta was quantified and statistically analysed (n = three mice per group). **G** qPCR assay to evaluate the miRNA levels of miR159a, miR166a-3p and miR170-5p in aorta tissues. The graphs show the quantification of the indicated miRNA normalized to U6 snRNA (n = five mice per group). Data represent means ± SD. *P < 0.05, **P < 0.01, ***P < 0.001

While EVs are typically administered via intravenous or direct injection into target organs/tissues, these delivery methods can be inconvenient, limiting their therapeutic application [21]. In contrast, PDNVs have the advantage of oral absorption, which may improve accessibility and expand their potential therapeutic use. We administered DiR dye-labeled CDNVs (DiR-CDNVs) to male C57BL/6 mice via gavage and observed their biodistribution outside the gastrointestinal tract. We found that DiR-CDNVs entered the bloodstream and accumulated in the liver, lungs, heart, and aorta, with low accumulation in the spleen and kidneys (Fig. 2A). To confirm that DiR-CDNVs accumulated in the heart and aorta, we used PBS and DiR dye gavage as controls and found that only DiR-CDNVs exhibited a strong fluorescent signal in the heart and aorta (Fig. 2B-C). We prepared tissue sections of the aorta and observed that DiR-CDNVs accumulated in the vascular wall, consistent with our ex vivo imaging results (Fig. 2D). Furthermore, increasing the concentration of DiR-CDNVs increased the intensity of the DiR signal in the heart and aorta, which plateaued at 40 mg/kg (Fig. 2E). We also found that miR159a and miR166a-3p were significantly upregulated in the aortic tissues of the CDNV gavage group (Fig. 2F). These results suggest that CDNVs and their miRNAs can be absorbed through the gastrointestinal tract and taken up by the aortic tissue, providing a basis for their role in regulating the cardiovascular system.

**Fig. 3:**
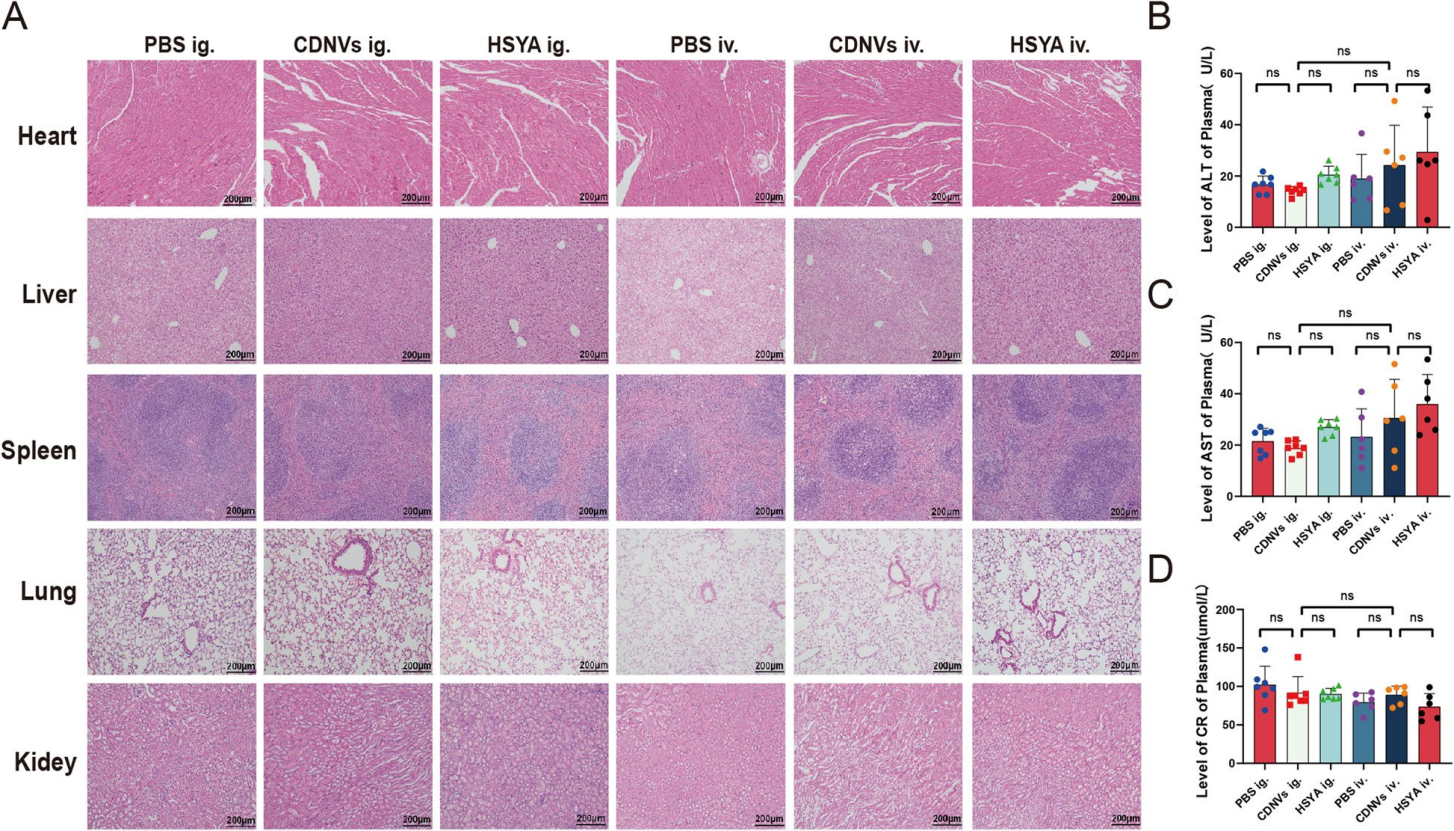
In vivo toxicity of CDNVs. **A** Representative images of H&E-stained liver, kidney, heart, lung and spleen sections from the indicated groups (n = six mice per group). Scale bar represents 200 μm. **B**-**D** The levels of liver injury-related biomarkers (serum ALT and AST) and kidney injury-related biomarkers (serum creatinine (CR)) in the indicated groups (n = six mice per group). Data represent means ± SD. ns, not significant. The details for each group were shown as below: PBS ig.: mice were orally administered with 200 µL of PBS; CDNVs ig.: mice were orally administered with 40 mg/kg of CDNVs; HSYA ig.: mice were orally administered with 0.27 mg/kg HSYA; PBS iv.: mice were intravenously administered with 200 µL of PBS; CDNVs iv.: mice were intravenously administered with 40 mg/kg of CDNVs; HSYA iv.: mice were intravenously administered with 0.27 mg/kg HSYA. All treatments were administered three times per week for 12 weeks. The H&E staining and the measurement of serum biomarkers were performed at the end of the 12-week treatment period

AS is a chronic systemic disease requiring long-term and frequent medication, making drug safety a crucial consideration in its management. Here, we aimed to evaluate the long-term safety of CDNVs in ApoE-/- mice regarding organ morphology and liver and kidney injury after administration. CDNV administration did not result in tissue damage, and serum analysis showed no significant changes in ALT, AST, or CR levels (Fig. 3A). These findings suggest that CDNVs are safe and non-toxic, regardless of the route of administration.

### CDNVs reduce the plaque burden in ApoE-/- mice

**Fig. 4:**
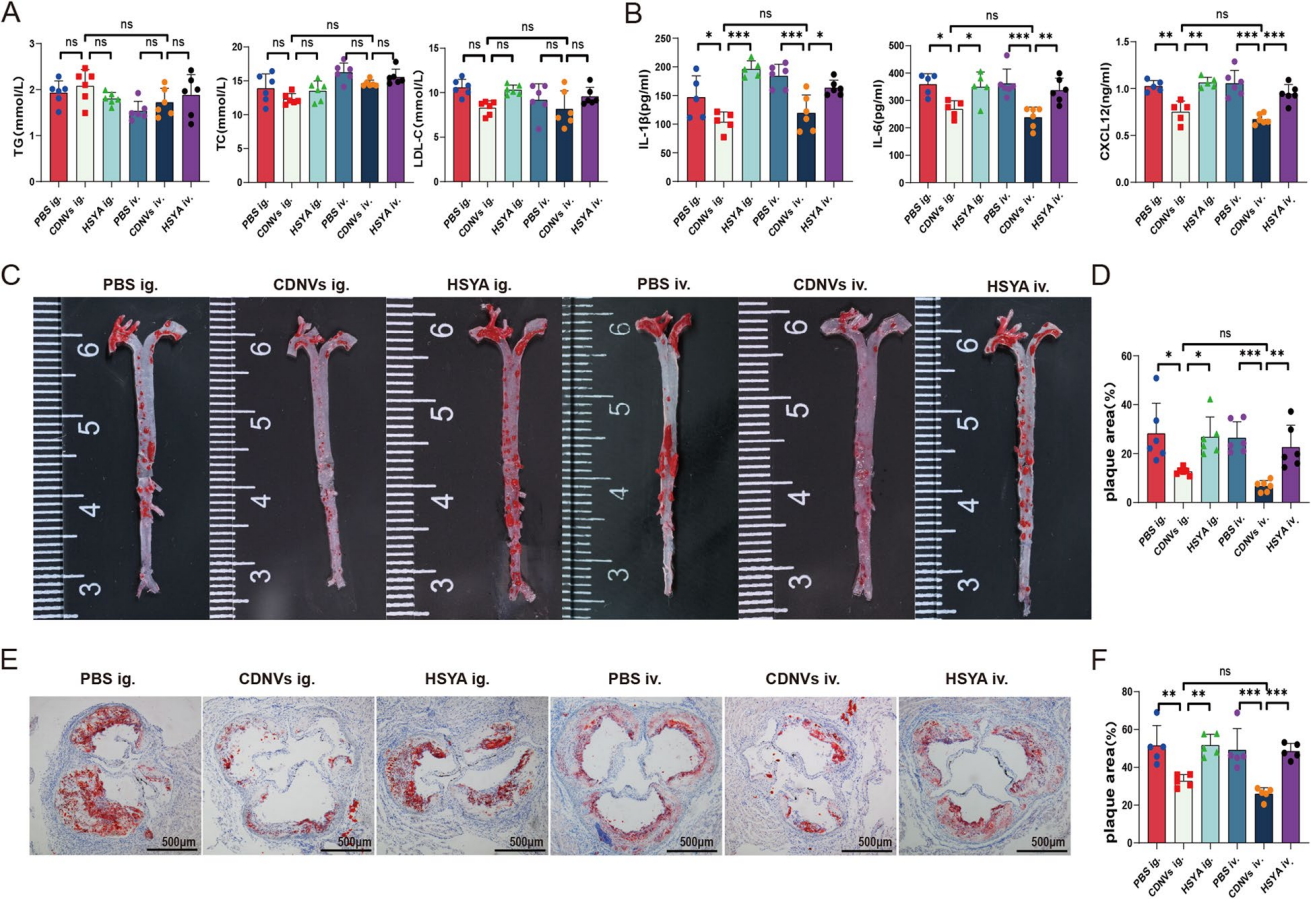
Atheroprotective effect of CDNVs. **A** The serum levels of triglycerides (TG), total cholesterol (TC), and low-density lipoprotein cholesterol (LDL-C) were measured in the indicated groups of mice (n = six mice per group). **B** The serum levels of interleukin-1β (IL-1β), interleukin-6 (IL-6), and CXCL12 were measured in the indicated groups of mice (n = six mice per group). **C** Oil-red-O staining was used to visualize atherosclerotic plaques in the aorta trees of ApoE-/- mice, and representative images were taken. **D** The area of atherosclerotic plaques in the aorta trees of ApoE-/- mice was quantified and statistically analysed (n = six mice per group). **E** Oil-red-O staining was used to visualize atherosclerotic plaques in the aorta roots of ApoE-/- mice, and representative images were taken. **F** The area of atherosclerotic plaques in the aorta roots of ApoE-/- mice was quantified and statistically analysed (n = five mice per group). Scale bar represents 500 μm. Data are presented as the means ± SD. Significance levels are indicated as *P < 0.05, **P < 0.01, ***P < 0.001, or ns (not significant). The details for each group were shown as below: PBS ig.: mice were orally administered with 200 µL of PBS; CDNVs ig.: mice were orally administered with 40 mg/kg of CDNVs; HSYA ig.: mice were orally administered with 0.27 mg/kg HSYA; PBS iv.: mice were intravenously administered with 200 µL of PBS; CDNVs iv.: mice were intravenously administered with 40 mg/kg of CDNVs; HSYA iv.: mice were intravenously administered with 0.27 mg/kg HSYA. All treatments were administered three times per week for 12 weeks. The H&E staining and the measurement of serum biomarkers were performed at the end of the 12-week treatment period

After the 12-week high-fat diet, mice were assessed to determine the effects of CDNVs on lipid metabolism, inflammation, and the burden of atherosclerotic plaques. The results revealed no significant differences in body weight or serum lipid levels among the groups, indicating that CDNVs had no substantial impact on lipid metabolism in mice (Additional file 1: Fig. S4A, Fig. 4A). However, CDNVs significantly reduced the levels of IL-1β, IL-6, and CXCL12, which are inflammation biomarkers [24] (Fig. 4B). The burden of atherosclerotic plaques was evaluated by measuring the plaque area in the aorta trees and aortic roots. CDNV treatment led to a significant reduction in the burden of atherosclerotic plaques (Fig. 4C-F). Furthermore, we observed that oral administration of CDNVs was as effective as intravenous injection. This suggests that oral administration is a viable and convenient delivery route for CDNVs. Additionally, HSYA did not have a significant effect on inflammatory cytokine levels or plaque burden, indicating that CDNVs were more effective than HSYA. These results suggest that CDNVs may offer a more pronounced anti-atherosclerotic benefit than HSYA and that this benefit is not solely dependent on the presence of HSYA.

### Protective effects of CDNVs on ox-LDL-treated HUVECs

**Fig. 5:**
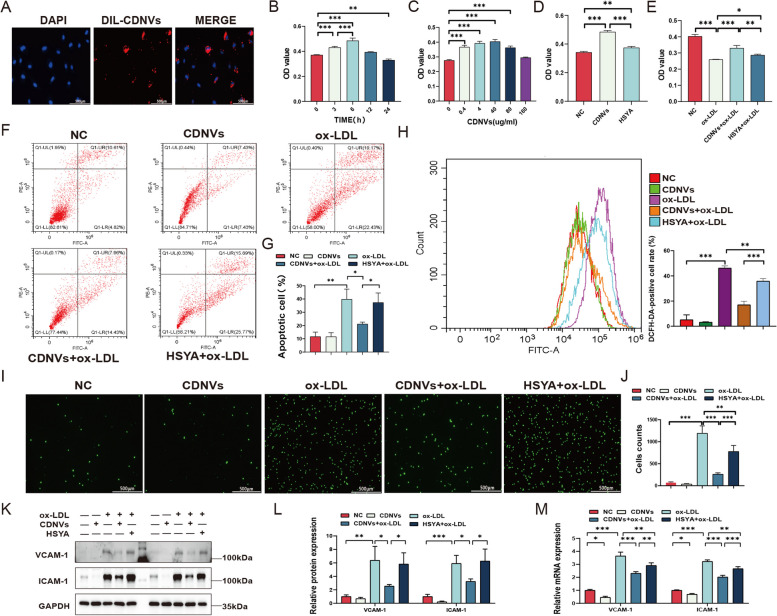
Protective effect of CDNVs in ox-LDL-treated HUVECs. **A** Confocal microscopy images were used to show the uptake of CDNVs by HUVECs, with a scale bar representing 100 μm. **B** The effect of CDNVs (40 µg/ml) on HUVEC proliferation was evaluated using a CCK-8 assay at different treatment durations. **C** The effect of CDNVs (0, 0.4, 4, 40, 80 and 160 µg/ml) on HUVEC proliferation was evaluated at different concentrations using a CCK-8 assay. **D** The effect of CDNVs (40 µg/ml) and HSYA (0.27 µg/ml) on HUVEC proliferation was compared using a CCK-8 assay. **E** The effect of CDNVs (40 µg/ml) and HSYA (0.27 µg/ml) on the proliferation of ox-LDL-treated (100 µg/ml) HUVECs was compared using a CCK-8 assay. **F**-**G** The effect of CDNVs (40 µg/ml) and HSYA (0.27 µg/ml) on ox-LDL-induced (100 µg/ml) apoptosis in HUVECs was evaluated and statistically analysed. **H** The effect of CDNVs (40 µg/ml) and HSYA (0.27 µg/ml) on the level of ROS in ox-LDL-treated (100 µg/ml) HUVECs was measured. **I**-**J** Fluorescence microscopy images were used to show the adhesion of monocytes to HUVECs, with a scale bar representing 500 μm, and the results were statistically analysed. **K** The levels of VCAM-1 and ICAM-1 proteins in HUVECs were analysed using Western blotting. **L** The levels of VCAM-1 and ICAM-1 proteins in HUVECs, as normalized to GAPDH, were quantified. **M** qPCR assays were conducted to measure the mRNA levels of VCAM-1 and ICAM-1 in HUVECs, and the results were quantified and normalized to GAPDH. The results represent three independent experiments (*n* = 3). Data represent means ± SD. *P < 0.05, **P < 0.01, ***P < 0.001

Subsequently, we examined the uptake and biological effects of CDNVs in HUVECs. CDNVs were labeled with DiL and DiO dyes and co-cultured with HUVECs for uptake evaluation using confocal microscopy and flow cytometry. Our results demonstrated that the uptake of CDNVs by HUVECs was time-dependent, reaching an uptake rate of approximately 50% at 6 h (Fig. 5A, Additional file 1: Fig. S5A-B).

To assess the impact of CDNVs on HUVEC proliferation, we conducted the CCK-8 assay with varying concentrations and incubation times. The results indicated that CDNVs exerted a concentration-dependent and time-dependent effect on HUVEC proliferation, with 40 µg/ml and 6 h representing the optimal conditions (Fig. 5B). When comparing the proliferative effects of HSYA and CDNVs, it was observed that while HSYA modestly promoted HUVEC proliferation, its effect was significantly weaker compared to CDNVs (Fig. 5D). Additionally, we examined the effects of CDNVs and HSYA on the proliferation of ox-LDL-treated HUVECs. Our results revealed that CDNVs significantly mitigated the inhibitory effect of ox-LDL on HUVEC proliferation, whereas HSYA had only a minor effect compared to CDNVs (Fig. 5E).

The development of atherosclerosis (AS) is strongly associated with endothelial cell apoptosis and the activation of reactive oxygen species (ROS), which impair endothelial barrier function and promote plaque formation [25, 26]. Hence, we investigated the potential of CDNVs as therapeutic agents for AS by evaluating their effects on ox-LDL-induced apoptosis and ROS activation in HUVECs. The results demonstrated that CDNVs exhibited high efficacy in reducing endothelial cell apoptosis and oxidative stress induced by ox-LDL, while HSYA exhibited a relatively weaker effect (Fig. 5F-H).

AS is a pathogenic process characterized by the adhesion of monocytes to endothelial cells in response to endothelial injury [27, 28]. Monocyte adhesion assays are commonly used to assess the adhesion ability of endothelial cells and the extent of inflammatory response [27]. Notably, pretreatment with CDNVs effectively reduced monocyte adhesion to endothelial cells, surpassing the effects of HSYA (Fig. 5I-J). VCAM-1 and ICAM-1 are cell adhesion molecules that play pivotal roles in mediating the adhesion of inflammatory cells such as monocytes, lymphocytes, eosinophils, and basophils to the vascular endothelium, thereby exacerbating endothelial inflammation [29]. Consequently, they are commonly used as markers to evaluate the inflammatory status of endothelial cells. Western blotting and qPCR analyses revealed that ox-LDL treatment significantly upregulated the expression of VCAM-1 and ICAM-1 in HUVECs, while pretreatment with CDNVs markedly attenuated the ox-LDL-induced increase in VCAM-1 and ICAM-1 expression, demonstrating a superior effect compared to HSYA (Fig. 5K-M).

### miRNA and mRNA sequencing of CDNV-treated HUVECs

**Fig. 6:**
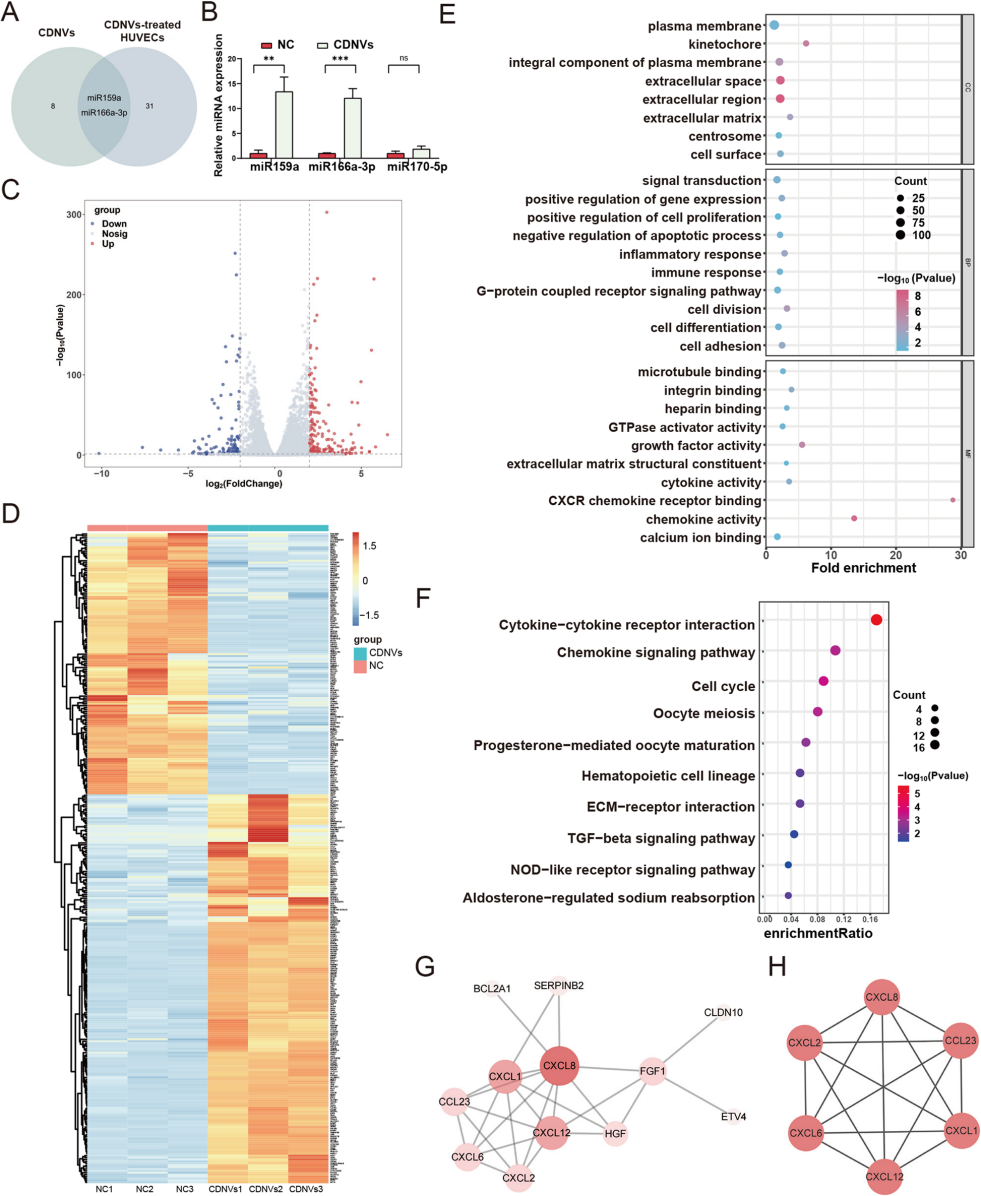
RNA sequencing analysis and bioinformatics of CDNVs-treated HUVECs. **A** Venn diagrams were used to analyse the intersection of upregulated miRNAs in HUVECs after CDNV intervention and miRNAs contained in CDNVs. **B** qPCR assays were conducted to measure the miRNA levels of miR159a, miR166a-3p, and miR170-5p in HUVECs treated with or without CDNVs, and the results were quantified and normalized to U6 snRNA. The results represent three independent experiments (*n* = 3). Data are presented as the means ± SD. Significance levels are indicated as **P < 0.01, ***P < 0.001, or ns (not significant). **C** Volcano plots were used to visualize the differentially expressed genes (DEGs) in HUVECs after CDNV treatment, with red dots indicating upregulated genes, blue dots indicating downregulated genes, and gray dots indicating genes with no significant changes. **D** A heatmap was used to display the expression patterns of DEGs in HUVECs after CDNV treatment, with the color scale representing the relative expression level, with red indicating upregulation and blue indicating downregulation. **E** GO enrichment analysis was conducted to identify the biological processes, molecular functions, and cellular components that were enriched among the DEGs in HUVECs. **F** KEGG pathway analysis was performed to identify the pathways that were significantly enriched among the DEGs in HUVECs. **G** A PPI network was constructed to visualize the interactions among the DEGs in HUVECs after CDNV treatment. The circles vary in color from dark to light, representing the degree from high to low. **H** Hub genes were identified among the DEGs in the PPI network using the MCODE module

To investigate the delivery of CDNV-miRNAs into HUVECs, we performed small RNA sequencing with and without CDNV co-culture. The results revealed significant changes in 60 miRNAs, with 33 upregulated and 27 downregulated. Intersectional analysis of CDNV-miRNAs and upregulated miRNAs in CDNV-treated HUVECs identified two overlapping miRNAs: miR159a and miR166a-3p (Fig. 6A). Furthermore, qPCR confirmed the significant upregulation of these miRNAs in the cells, while the expression of miR170-5p, which was not an overlapping miRNA, remained unchanged (Fig. 6B). These findings indicate that CDNVs can deliver miRNAs to HUVECs.

Transcriptome sequencing was performed on HUVECs with and without CDNV treatment. The results revealed 341 genes with significant differential expression, including 202 upregulated and 139 downregulated genes (Fig. 6C-D). GO enrichment analysis of these differentially expressed genes (DEGs) showed their involvement in cellular components such as the kinetochore and extracellular region, while molecular functions included chemokine activity, CXCR chemokine receptor binding, and cytokine activity. Biological process enrichment included inflammatory response, immune response, and cell adhesion (Fig. 6E). According to KEGG pathway analysis, the DEGs were mainly enriched in cytokine-cytokine receptor interactions and the chemokine signaling pathway (Fig. 6F). Additionally, a PPI network consisting of 12 nodes was constructed for the DEGs. Consistent with the KEGG pathway analysis, the genes in the PPI network were mainly associated with inflammation, including genes from the chemokine family such as CXCL1, CXCL2, CXCL6, CXCL8, CXCL12, and CCL23, as well as other inflammation-related genes like CRP, HGF, and FGF1 [30] (Fig. 6G). Furthermore, CXCL1, CXCL2, CXCL6, CXCL8, CXCL12, and CCL23 were identified as hub genes enriched by the MCODE algorithm (Fig. 6H). These genes are widely involved in mediating inflammatory responses and are closely related to the development of AS [31–33]. These findings suggest that the protective effects of CDNVs on endothelial cells are related to the regulation of inflammation.

### CXCL12 as a potential direct target of miR166a-3p

**Fig. 7:**
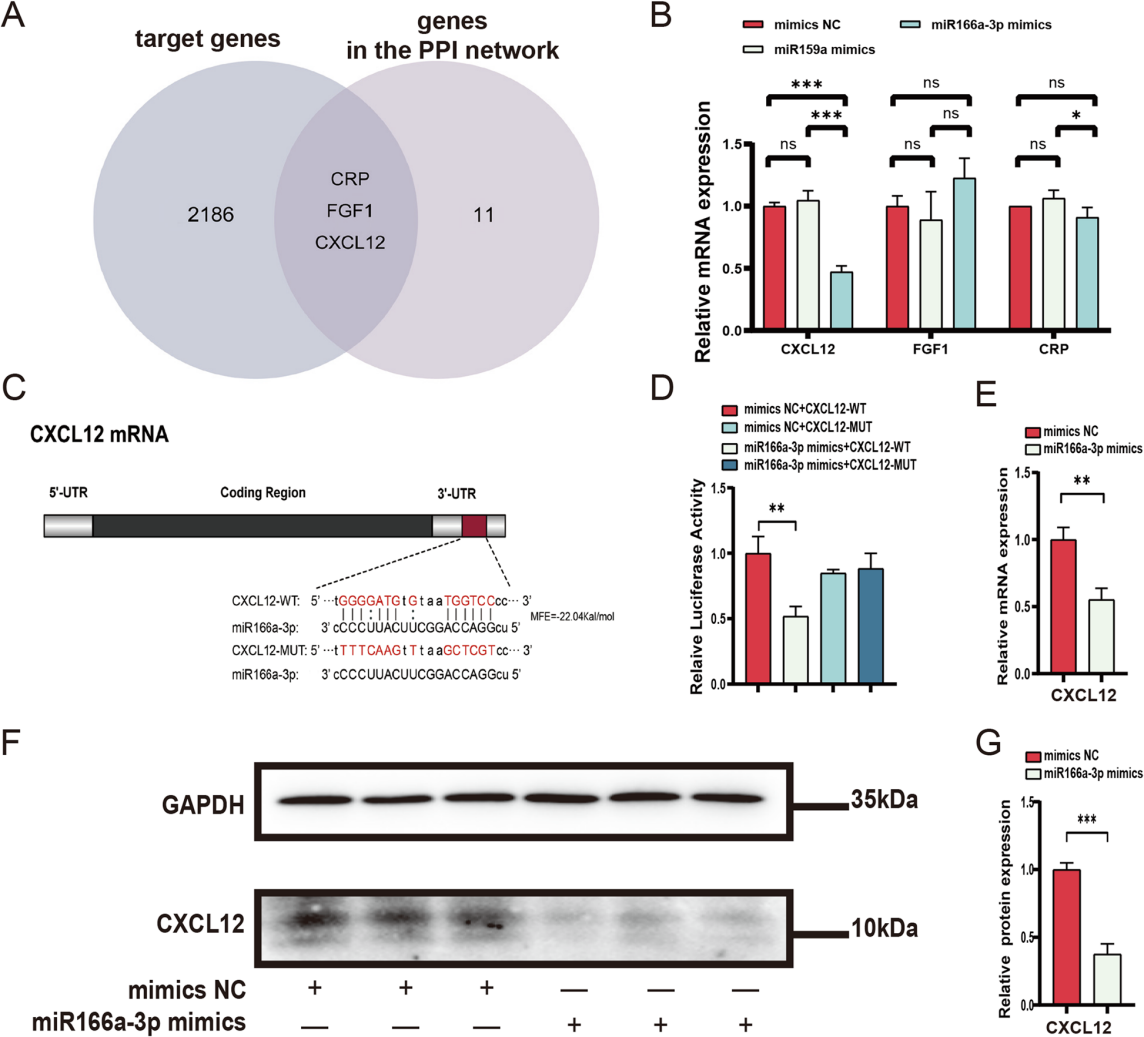
CXCL12 is predicted to be a direct target of miR166a-3p. **A** Venn diagrams were used to analyse the intersection of target genes of miRNAs in CDNVs and genes in the PPI network. **B** qPCR assays were conducted to measure the mRNA levels of CXCL12, CRP, and FGF1 in HUVECs, and the results were quantified and normalized to GAPDH. **C** A schematic description of the hypothesized duplexes formed by interactions between the 3’UTR of human CXCL12 and miR166a-3p is shown, with the predicted maximum free energy (MFE) of the hybrid indicated. A CXCL12-MUT plasmid was constructed, and the mutated nucleotides are shown in red. **D** Dual-luciferase reporter gene assays were performed to confirm the interaction between miR166a-3p and CXCL12. **E** qPCR assays were conducted to measure the mRNA levels of CXCL12 in HUVECs, and the results were quantified and normalized to GAPDH. **F** Western blot assays were conducted to measure the protein expression levels of CXCL12 in HUVECs, and the results were quantified and normalized to GAPDH. **G** Quantification of the protein expression levels of CXCL12 in HUVECs, as normalized to GAPDH, is shown. The results represent three independent experiments (*n* = 3). Data represent means ± SD. *P < 0.05, **P < 0.01, ***P < 0.001

Our next objective was to identify key genes closely related to CDNV-miRNAs among the DEGs. Specifically, we conducted gene intersection experiments between the target genes of miR159a and miR166a-3p and the genes in the PPI network. This intersection revealed CXCL12, CRP, and FGF1 as the genes (Fig. 7A). We selected these genes to validate the regulatory effect of miR159a and miR166a-3p, which were successfully delivered into HUVECs by CDNVs. qPCR results demonstrated that miR166a-3p mimics significantly reduced the expression levels of CXCL12 mRNA (Fig. 7B). Furthermore, we used miRanda software to perform dynamic programming alignment between the 3’ UTR sequences of human CXCL12 and miR166a-3p (Fig. 7C). These results indicate that CXCL12 is a potential specific target of miR166a-3p. Subsequently, we constructed reporter plasmids with CXCL12-wild type and CXCL12-mutant type vectors (Fig. 7C). These plasmids were transfected into HEK-293T cells using either the miR166a-3p mimic or mimic NC. The results demonstrated that miR166a-3p mimics significantly reduced the luciferase activity of the CXCL12-WT plasmids, while the binding-site mutation did not have this effect (Fig. 7D), suggesting that miR166a-3p can specifically bind to transcripts of CXCL12.

To further validate whether this binding relationship caused changes in target gene expression, we performed qPCR experiments, which showed that the miR166a-3p mimics significantly inhibited the mRNA levels of CXCL12 in HUVECs (Fig. 7E). Moreover, western blot analysis demonstrated that the miR166a-3p mimic significantly inhibited the protein level of CXCL12 in HUVECs (Fig. 7F). These results suggest that CXCL12 may be a direct target of miR166a-3p. miR166a-3p is one of the two miRNAs that can be delivered into aortic tissue and HUVECs by CDNVs and has binding sites with the key gene CXCL12, indicating that miR166a-3p may be the most critical miRNA involved in the biological functions of CDNVs.

### CDNVs regulate HUVEC inflammation via the miR166a-3p/CXCL12 pathway

To elucidate the regulatory role of the miR166a-3p/CXCL12 pathway in HUVEC inflammation, we conducted rescue experiments to investigate the effects of this pathway on inflammation regulation in HUVECs. These experiments involved overexpressing and inhibiting miR166a-3p (Additional file 1: Fig. S6A–C) as well as modulating CXCL12 expression through overexpression and knockdown (Additional file 1: Fig. S6D–G).

**Fig. 8:**
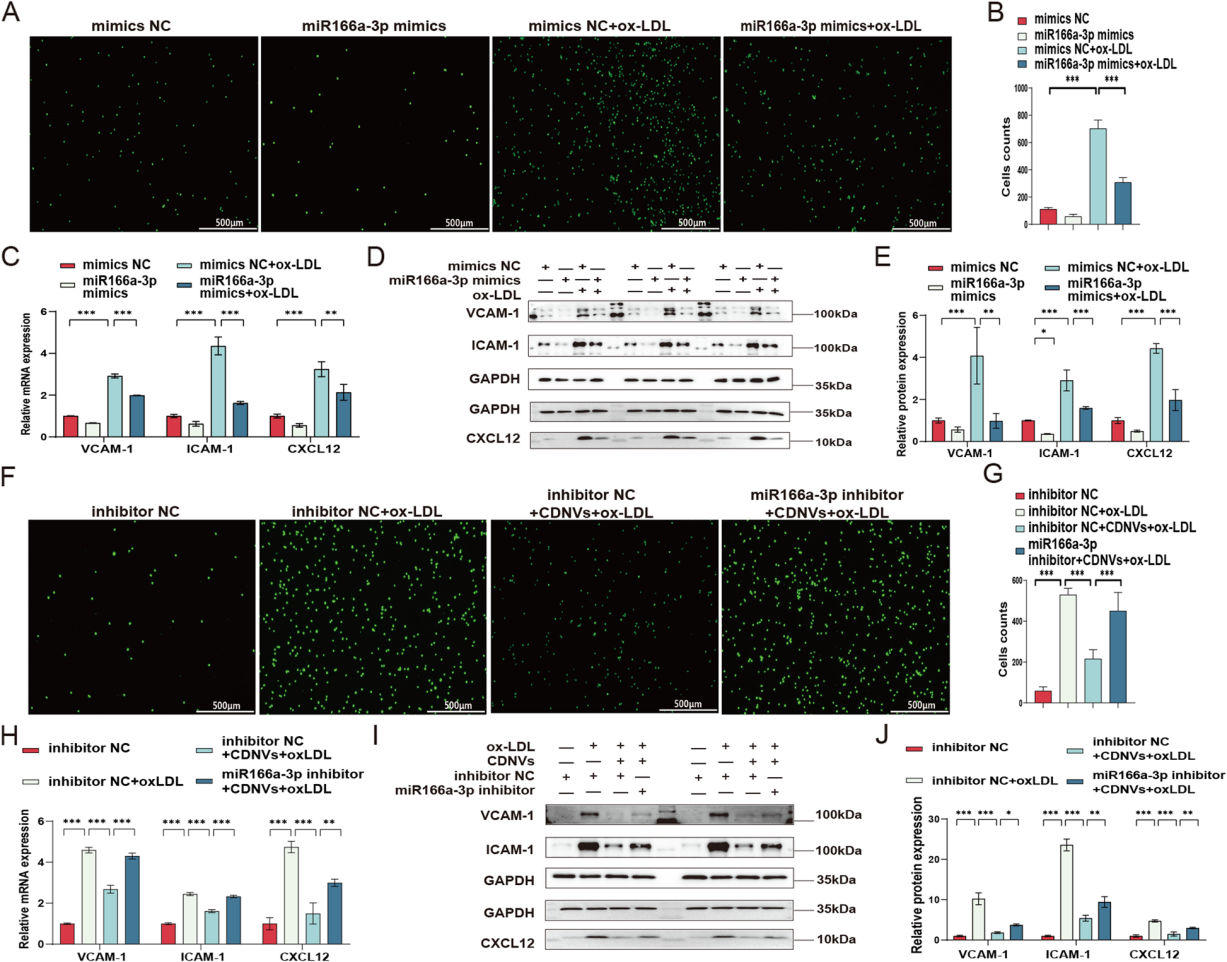
The role of miR166a-3p in CDNV-mediated attenuation of ox-LDL-induced inflammation in HUVECs. **A**-**B** Monocyte adhesion assays were performed, and statistical analysis was conducted. Scale bar: 500 μm. **C** qPCR assays were conducted to measure the mRNA levels of VCAM-1, ICAM-1 and CXCL12 in HUVECs, and the results were quantified and normalized to GAPDH. **D** Western blot assays were conducted to measure the protein expression levels of VCAM-1, ICAM-1 and CXCL12 in HUVECs. **E** Quantification of the protein expression levels of VCAM-1, ICAM-1 and CXCL12 in HUVECs, as normalized to GAPDH, is shown. **F**-**G** Monocyte adhesion assays were performed, and statistical analysis was conducted. Scale bar: 500 μm. **H** qPCR assays were conducted to measure the mRNA levels of VCAM-1, ICAM-1 and CXCL12 in HUVECs, and the results were quantified and normalized to GAPDH. **I** Western blot assays were conducted to measure the protein expression levels of VCAM-1, ICAM-1 and CXCL12 in HUVECs. **J** Quantification of the protein expression levels of VCAM-1, ICAM-1 and CXCL12 in HUVECs, as normalized to GAPDH, is shown. The results represent three independent experiments (*n* = 3). Data represent means ± SD. Significance levels are indicated as *P < 0.05, **P < 0.01, ***P < 0.001

Initially, we increased the levels of miR166a-3p in HUVECs using miR166a-3p mimics and observed that they alleviated ox-LDL-induced inflammation. This was evident by reduced monocyte adhesion and decreased expression of VCAM-1, ICAM-1, and CXCL12 at the mRNA and protein levels (Fig. 8A–E). However, this protective effect was not significant in unstimulated HUVECs. Subsequently, we employed a miR166a-3p inhibitor to counteract the influence of CDNVs on inflammation regulation in HUVECs. The results demonstrated that this inhibition negated the protective effects of CDNVs, as indicated by increased monocyte adhesion and elevated expression of VCAM-1, ICAM-1, and CXCL12 at both the mRNA and protein levels (Fig. 8F–J).

**Fig. 9:**
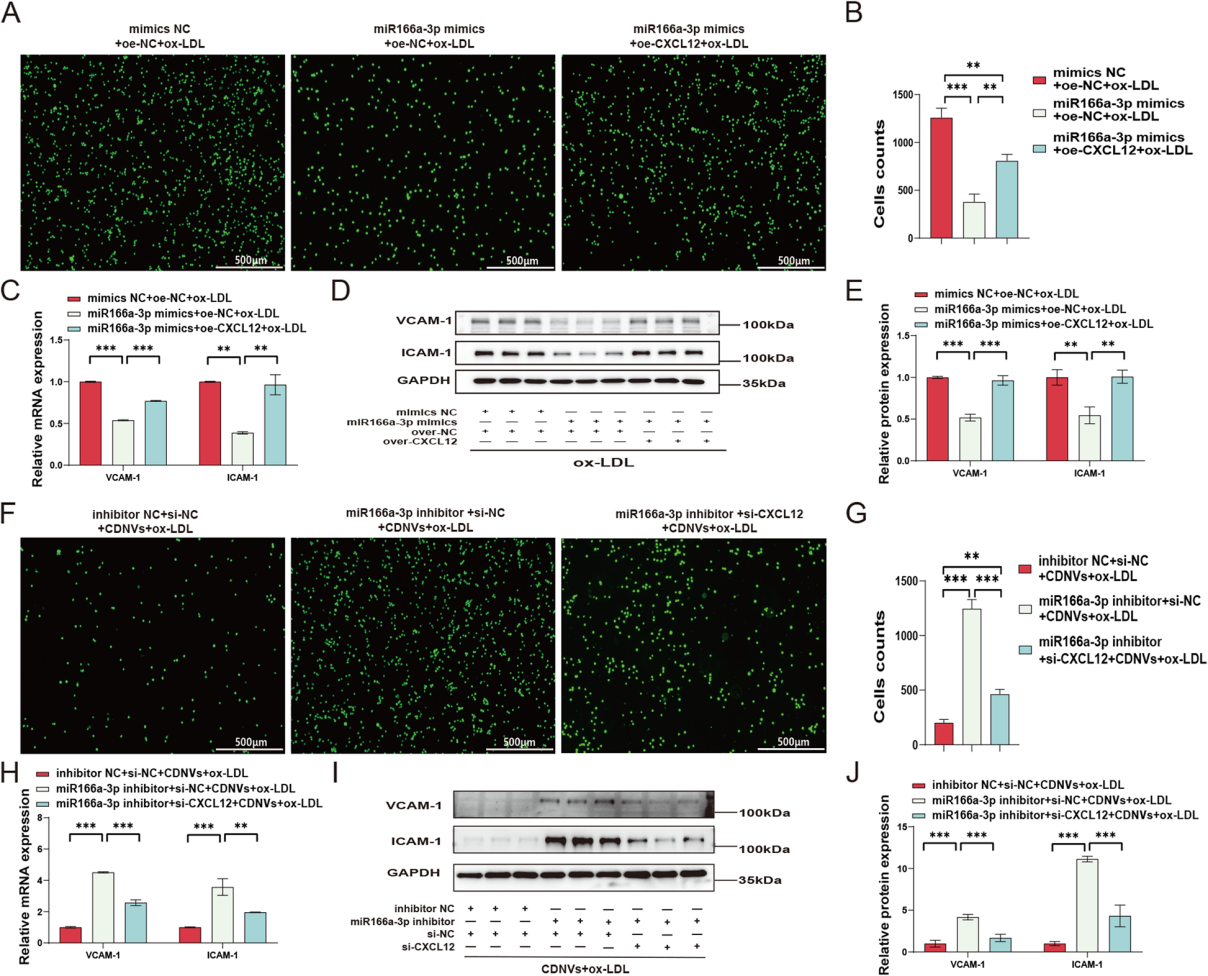
CDNVs regulate HUVEC inflammation via the miR166a-3p/CXCL12 pathway. **A**-**B** Monocyte adhesion assays were performed, and statistical analysis was conducted. Scale bar: 500 μm. **C** qPCR assays were conducted to measure the mRNA levels of VCAM-1 and ICAM-1 in HUVECs, and the results were quantified and normalized to GAPDH. **D** Western blot assays were conducted to measure the protein expression levels of VCAM-1 and ICAM-1 in HUVECs. **E** Quantification of the protein expression levels of VCAM-1 and ICAM-1 in HUVECs, as normalized to GAPDH, is shown. **F**-**G** Monocyte adhesion assays were performed, and statistical analysis was conducted. Scale bar: 500 μm. **H** qPCR assays were conducted to measure the mRNA levels of VCAM-1 and ICAM-1 in HUVECs, and the results were quantified and normalized to GAPDH. **I** Western blot assays were conducted to measure the protein expression levels of VCAM-1 and ICAM-1 in HUVECs. **J** Quantification of the protein expression levels of VCAM-1 and ICAM-1 in HUVECs, as normalized to GAPDH, is shown. The results represent three independent experiments (*n* = 3). Data represent means ± SD. Significance levels are indicated as *P < 0.05, **P < 0.01, ***P < 0.001

To further understand the contribution of CXCL12 to the effects of miR166a-3p, we constructed an adenovirus for CXCL12 overexpression (oe-CXCL12) and used siRNA for CXCL12 knockdown (si-CXCL12). The overexpression of CXCL12 counteracted the protective effect of miR166a-3p mimics, resulting in increased monocyte adhesion and elevated expression of VCAM-1 and ICAM-1 at the mRNA and protein levels (Fig. 9A–E). Conversely, si-CXCL12 reversed the antagonistic effect of the miR166a-3p inhibitor on CDNVs, leading to reduced monocyte adhesion and decreased expression of VCAM-1 and ICAM-1 at the mRNA and protein levels (Fig. 9F–J). Collectively, these results suggest that the regulatory effect of CDNVs on HUVEC inflammation largely depends on the delivery of miR166a-3p and that CXCL12 plays a critical role in the miR166a-3p pathway.

### Effect of miR166a-3p on AS in ApoE-/- mice

**Fig. 10:**
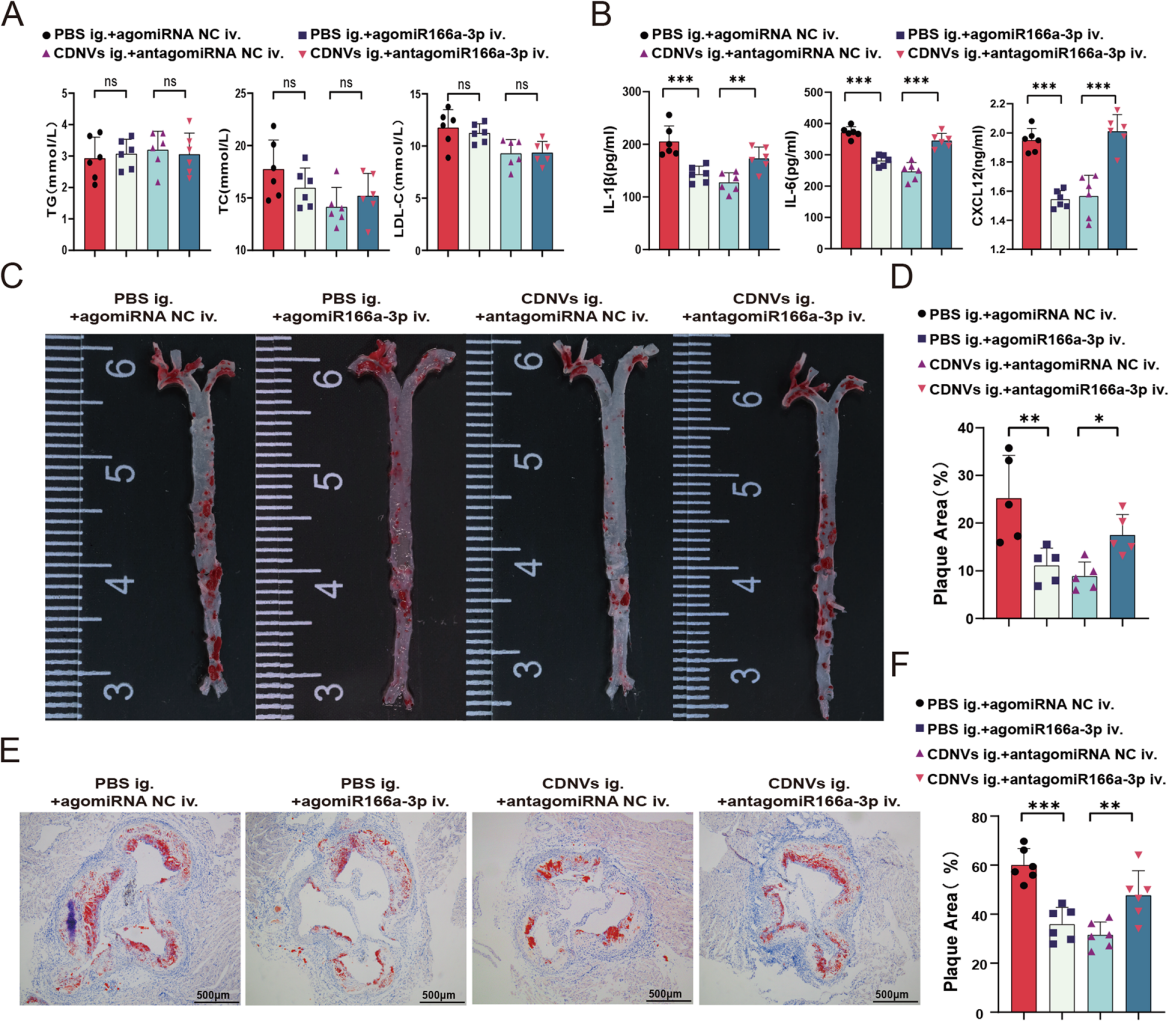
The effect of miR166a-3p on atherosclerosis in ApoE-/- mice. **A** The levels of serum TG, TC and LDL-C were measured in six mice per group. **B** The levels of serum IL-1β, IL-6, and CXCL12 were measured in six mice per group. **C** Representative images of oil-red-O staining of aortic trees in ApoE-/- mice were obtained. **D** Statistical analyses of plaque area in the aortic trees were performed using samples from five mice per group. **E** Representative images of oil-red-O staining of aortic roots in ApoE-/- mice were obtained. Scale bar: 500 μm. **F** Statistical analyses of plaque area in the aortic roots were performed using samples from six mice per group. Data are presented as the means ± SD. Significance levels are indicated as *P < 0.05, **P < 0.01, ***P < 0.001, or ns for not significant

To explore the potential effects of miR166a-3p derived from CDNVs in ApoE-/- mice fed a high-fat diet, we developed in vivo miR166a-3p mimics (agomiR166a-3p) and inhibitors (antagomiR166a-3p), and assessed their effects on body weight, lipid metabolism, inflammation, and atherosclerosis (AS). The results demonstrated that miR166a-3p had little effect on body weight or lipid metabolism (Additional file 1: Fig. S4B, Fig. 10A). However, administration of agomiR166a-3p produced anti-inflammatory effects similar to those of CDNVs, while miR166a-3p inhibitors significantly antagonized the anti-inflammatory effects of CDNVs (Fig. 10B). Moreover, continuous administration of agomiR166a-3p significantly alleviated the burden of atherosclerotic plaques in the aorta, whereas antagomiR166a-3p weakened the anti-atherosclerotic effects of CDNVs (Fig. 10C–F). These findings suggest that the anti-inflammatory and anti-atherosclerotic effects of CDNVs largely depend on the delivery of miR166a-3p in vivo.

## Discussion

In this study, we have demonstrated, for the first time, the remarkable anti-atherosclerotic effects of CDNVs. These effects can be attributed to the delivery of miR166a-3p into vascular tissues, which alleviates inflammation in endothelial cells. These results suggest that CDNVs hold promise as nanocarriers for delivering miRNAs to target vascular tissues and for the treatment of AS.

Research on PDNVs has primarily focused on inflammation-related digestive diseases, liver diseases, tumors, viral infections, and metabolic disorders, with few studies investigating their effects on cardiovascular diseases [34]. In this study, we isolated and purified CDNVs and investigated their biological functions for the first time. Chinese traditional medicine, *Carthamus tinctorius L* (CTL), is frequently used in the clinical treatment of conditions such as coronary heart and cerebrovascular diseases [35]. The extract from CTLs has been shown to enhance peripheral blood flow, inhibit platelet aggregation, and exhibit anti-atherosclerotic effects [16, 36]. The main active compound in the extract, HSYA, possesses strong anti-inflammatory and antioxidant effects [37]. However, the low oral bioavailability of HSYA limits the clinical application of CTLs to mainly injectable formulations, hindering their use in clinical practice. In contrast, we found that CDNVs demonstrate excellent anti-atherosclerotic effects, surpassing the efficacy of an equivalent dose of HSYA. Furthermore, our results showed that orally administered CDNVs have atheroprotective effects similar to those administered intravenously, suggesting that oral administration is a feasible approach for CDNV-based AS treatment. Moreover, the protective effects of CDNVs are relatively independent of HSYA, as other active ingredients likely contribute to their observed benefits.

MiRNAs are a class of single-stranded, noncoding small RNAs with a mature sequence length of approximately 20–24 nucleotide [38]. They regulate gene expression at the post-transcriptional level in mammals, plants, and microorganisms by binding to specific open reading frames or UTRs of mRNA, leading to cleavage or translation inhibition at the mRNA level [39]. Consequently, miRNAs play critical roles in post-transcriptional gene regulation in both plants and animals. In recent years, the biological functions of miRNAs derived from herbal medicines have garnered increasing interest due to a growing understanding of plant miRNAs. In 2015, Zhou et al. reported that miR2911 from honeysuckle remained stable in a honeysuckle decoction and could inhibit the replication of the H1N1 virus [40]. Subsequently, they found that miR2911 in a honeysuckle decoction effectively inhibited the replication of the SARS-CoV-2 virus in vivo, with administration of this decoction accelerating viral load reduction in patients with COVID-19 [41]. These studies have stimulated further exploration of the pharmacological effects of miRNAs in herbal medicine. For example, Qin et al. discovered that Sal-miR-58 from Salvia miltiorrhiza induces autophagy in human vascular smooth muscle cells (VSMCs) by targeting KLF3, thereby alleviating inflammation. Subsequent animal experiments have shown that artificially synthesized Sal-miR-58 inhibits the formation of abdominal aortic aneurysms induced by angiotensin II in mice [42]. Additionally, Xu et al. found that miRNAs present in nanovesicles derived from ginseng can stimulate stem cell differentiation both in vivo and in vitro, thereby promoting wound healing [43]. Recently, Han et al. extracted nanovesicles from the herbal medicine *Phellinus linteus* and investigated their effects on UV-induced skin aging. They discovered that miR-CM1 from *Phellinus linteus* could inhibit the expression of Mical2 in HaCaT cells through cross-kingdom regulation [44]. These findings demonstrate that miRNAs in herbal medicines can serve as active ingredients and exert biological effects. Our results are consistent with previous studies and highlight the distinct biological effects of miRNAs derived from the herbal medicine CTL. The direct regulation of CXCL12 by CDNVs through the delivery of miR166a-3p may be the most crucial mechanism underlying the anti-inflammatory and atheroprotective effects.

CXCL12, also known as stromal cell-derived factor-1, plays a role in regulating haematopoietic stem and progenitor cells in the bone marrow [45]. Recent research has shown a strong association between CXCL12 and AS. Genome-wide association studies of coronary artery disease and myocardial infarction have identified CXCL12 as a potential locus for AS [46, 47]. Clinical studies have also demonstrated a close relationship between CXCL12 and the severity of coronary artery disease. While CXCL12 is highly expressed in atherosclerotic plaques, it is expressed at low levels in normal blood vessels [48]. Additionally, CXCL12 is involved in various pathological processes of AS, including the regulation of foam and inflammatory cell formation, recruitment, and activation within the plaque, which contribute to accelerated plaque development [49–51]. Notably, a previous study highlighted the significant role of endothelial cell-derived CXCL12 as a potent pro-atherosclerotic molecule [52]. Given its strong pro-atherosclerotic effect, reducing circulating CXCL12 levels or inhibiting CXCL12 function may represent a promising therapeutic approach for the prevention and treatment of AS. Our study revealed that CXCL12 may be a direct target of miR166a-3p derived from CDNVs. Specifically, CDNVs may exert anti-inflammatory effects on endothelial cells by delivering miR166a-3p to HUVECs and targeting CXCL12. Furthermore, we confirmed that miR166a-3p can decrease serum CXCL12 levels in vivo and improve AS, suggesting that the atheroprotective effect of CDNVs is associated with the delivery of miR166a-3p. Although CXCL12 is a potential therapeutic target for AS, there are currently no targeted drugs available for clinical treatment. In this research, we present CDNVs as a safe and non-toxic vascular protective agent that can specifically regulate the expression of CXCL12.

However, our study has several limitations that should be noted. Firstly, we did not extensively investigate the digestion, absorption, and delivery of CDNVs to vascular tissues, including atherosclerotic plaque tissues. Further studies are needed to fully understand the pharmacokinetics and biodistribution of CDNVs in vivo. However, the complexity of PDNV absorption in the gastrointestinal tract, in vivo transport, and tissue targeting presents a significant challenge for further investigation. Secondly, we did not validate the regulatory role of miR166a-3p on CXCL12 in vivo, and the complete confirmation of whether miR166a-3p exerts anti-atherosclerotic effects through the regulation of CXCL12 is lacking. In the next phase of our research, we plan to investigate the role of the miR166a-3p/CXCL12 pathway in atherosclerosis by overexpressing or knocking out the CXCL12 gene in transgenic mice. Thirdly, although our focus was primarily on the role of miR166a-3p in endothelial cells, it should be acknowledged that other mechanisms involving macrophages and VSMCs also contribute to the development of atherosclerosis. Therefore, further studies will investigate the effects of CDNVs on these cell types. Fourthly, our experimental results also suggest that CDNVs are relatively homogeneous nanovesicles. However, due to current technological limitations, the majority of PDNV are still obtained through destructive processes, and it is not possible to completely eliminate artificial nanoparticles/microsomes and disrupted cellular membranes [21]. Although some studies suggest that certain proteins like PEN1, PEN3, and the Tetraspanin-8 may aid in the identification of PDNV [53], the roles of these proteins still require further research and may not necessarily apply to all plants. Thus, more research is needed to establish widely applicable specific markers for PDNVs.

## Conclusions

In conclusion, we successfully isolated and purified CDNVs that share similarities with mammalian extracellular vesicles. CDNVs possess membrane structures, exhibit relatively uniform size, and are rich in miRNAs. CDNVs are absorbed into the bloodstream through the gastrointestinal tract and display organ-selective distribution in vivo. The miRNAs within CDNVs can be taken up by vascular tissues through oral administration, where they exhibit a significant atheroprotective effect primarily mediated by miR166a-3p. Further exploration of the molecular mechanism revealed that CDNVs may exert an anti-inflammatory effect on ox-LDL-treated HUVECs through the miR166a-3p/CXCL12 pathway. Our results suggest that CDNVs are promising and innovative nanoplatforms for miRNA delivery in the treatment of AS. The findings of this study provide a foundation for the development of novel dosage forms of herbal medicines.

### Supplementary Information

**Additional file 1: Fig. S1.** CDNV purification and concentration of indicated ingredients in CDNVs. **Fig. S2.** Small RNA sequencing analysis and bioinformatics of CDNVs. **Fig. S3.** In vitro digestion of CDNVs. **Fig. S4.** Body weight of ApoE-/- mice. **Fig. S5.** Uptake efficiency of CDNVs by HUVECs. **Fig. S6.** Construction of miR166a-3p mimics, miR166a-3p inhibitor, oe-CXCL12, and si-CXCL12.

## Data Availability

The data that support the findings of this study are available from the corresponding authors upon reasonable request.

## Abbreviations

AS: Atherosclerosis
EVs: Extracellular vesicles
miRNAs: MicroRNAs
PDNVs: Plant derived nanovesicles
CTL: *Carthamus tinctorius L.*
HSYA: Hydroxysafflor yellow A
CDNVs: *Carthamus tinctorius**L.* Derived nanovesicles
TEM: Transmission electron microscopy
NTA: Nanoparticle Tracking Analysis
ig.: Intragastric
iv.: Intravenous
CE: Collision energy
CR: Creatinine
TG: Triglyceride
TC: Total cholesterol
LDL-C: Low-density lipoprotein cholesterol
ELISA: Enzyme-linked immunosorbent assay
CXCL12: Chemokine ligand 12
HUVECs: Human umbilical vein endothelial cells
VSMCs: Vascular smooth muscle cells
ox-LDL: Oxidized low density lipoprotein
ROS: Reactive oxygen species
qPCR: Quantitative real-time PCR
GO: Gene ontology
KEGG: Kyoto Encyclopedia of Genes and Genomes
DEGs: Differentially expressed genes
WT: Wild type
MUT: Mutant type
MFE: Max free energy
oe-CXCL12: CXCL12 overexpression
si-CXCL12: CXCL12 siRNA
VCAM-1: Vascular cell adhesion molecule-1
ICAM-1: Intercellular cell adhesion molecule-1
UTR: Untranslated region
