# Supplementary material for: Investigating the therapeutic effects and mechanisms of Carthamus tinctorius L.-derived nanovesicles in atherosclerosis treatment
Source: Cell Commun Signal. 2024 Mar 12;22:178. doi: 10.1186/s12964-024-01561-6 (PMC10936069; doi:10.1186/s12964-024-01561-6)
Supplement: Supplementary file 1 — Additional file 1: Fig. S1. CDNV purification and concentration of indicated ingredients in CDNVs. Fig. S2. Small RNA sequencing analysis and bioinformatics of CDNVs. Fig. S3. In vitro digestion of CDNVs. Fig. S4. Body weight of ApoE-/- mice. Fig. S5. Uptake efficiency of CDNVs by HUVECs. Fig. S6. Construction of miR166a-3p mimics, miR166a-3p inhibitor, oe-CXCL12, and si-CXCL12. [file 12964_2024_1561_MOESM1_ESM.doc]

**1. Supplementary Figures and Legends**

**
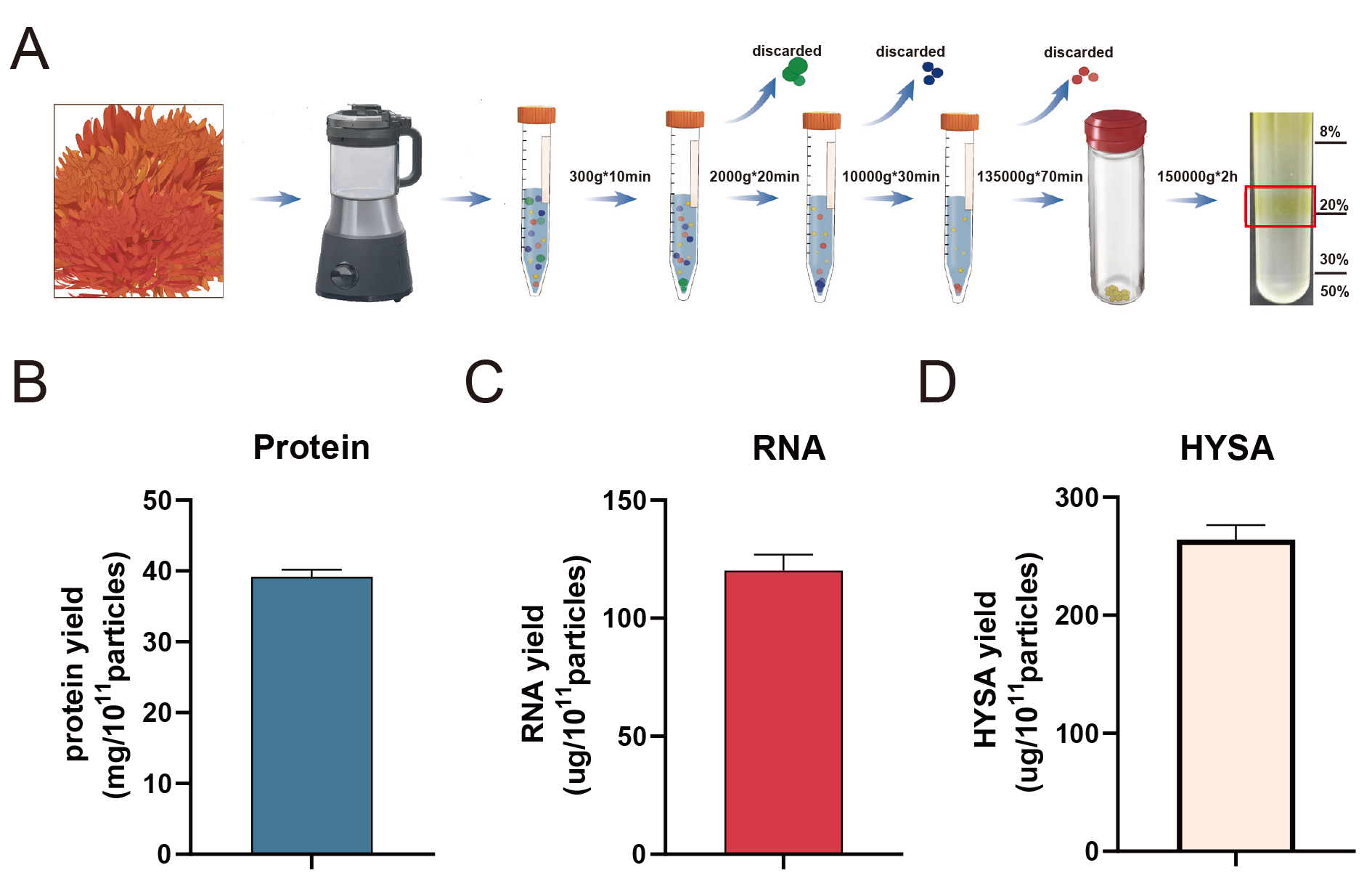
**

**Fig. S1 CDNV purification and concentration of the indicated ingredients in CDNVs.**

(A) Flow diagram illustrating the steps involved in the purification of CDNVs.

(B) Protein levels in CDNVs, as quantified using a BCA commercial kit. (C) Levels of RNA in CDNVs, as quantified using an Agilent 2100 Bioanalyzer. (D) Levels of HYSA in CDNVs, as quantified using UHPLC‒MS/MS. Results are presented as means ± SD from three independent experiments (n=3).


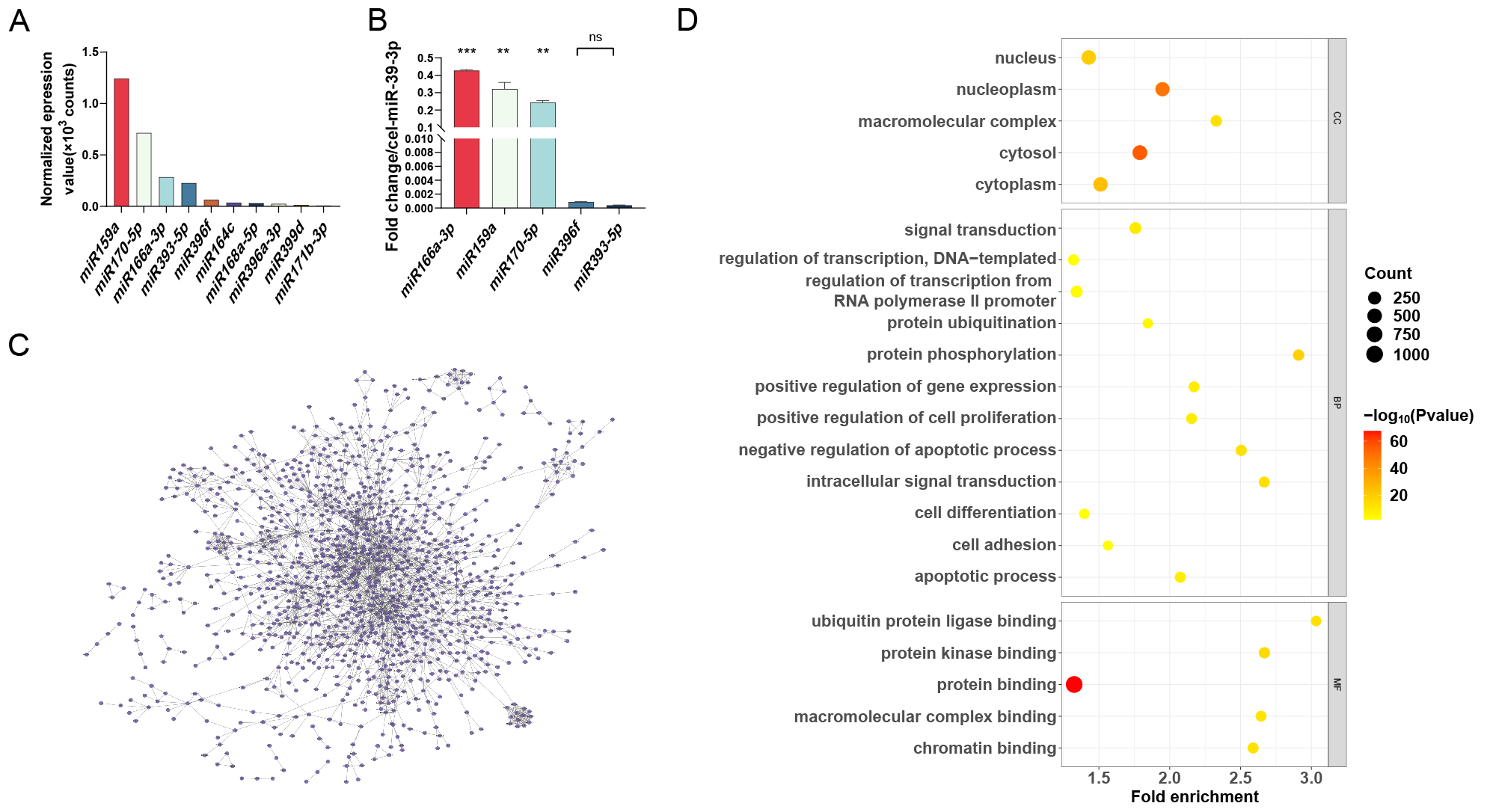


**Fig. S2 Small RNA sequencing analysis and bioinformatics of CDNVs.**

(A) Analysis of miRNAs commonly expressed in three batches of CDNV samples. (B) qPCR assay to evaluate the level of the top five miRNAs in CDNVs. The graphs show the quantification of the indicated miRNAs normalized to cel-miR-39-3p. Results are presented as means ± SD from three independent experiments (n=3). Data represent means ± SD. **P <0.01, ***P <0.001. ns, not significant. (C) PPI network of target genes of the top three miRNAs in CDNVs. (D) GO enrichment analysis of target genes in the PPI network of miRNAs in CDNVs.

**
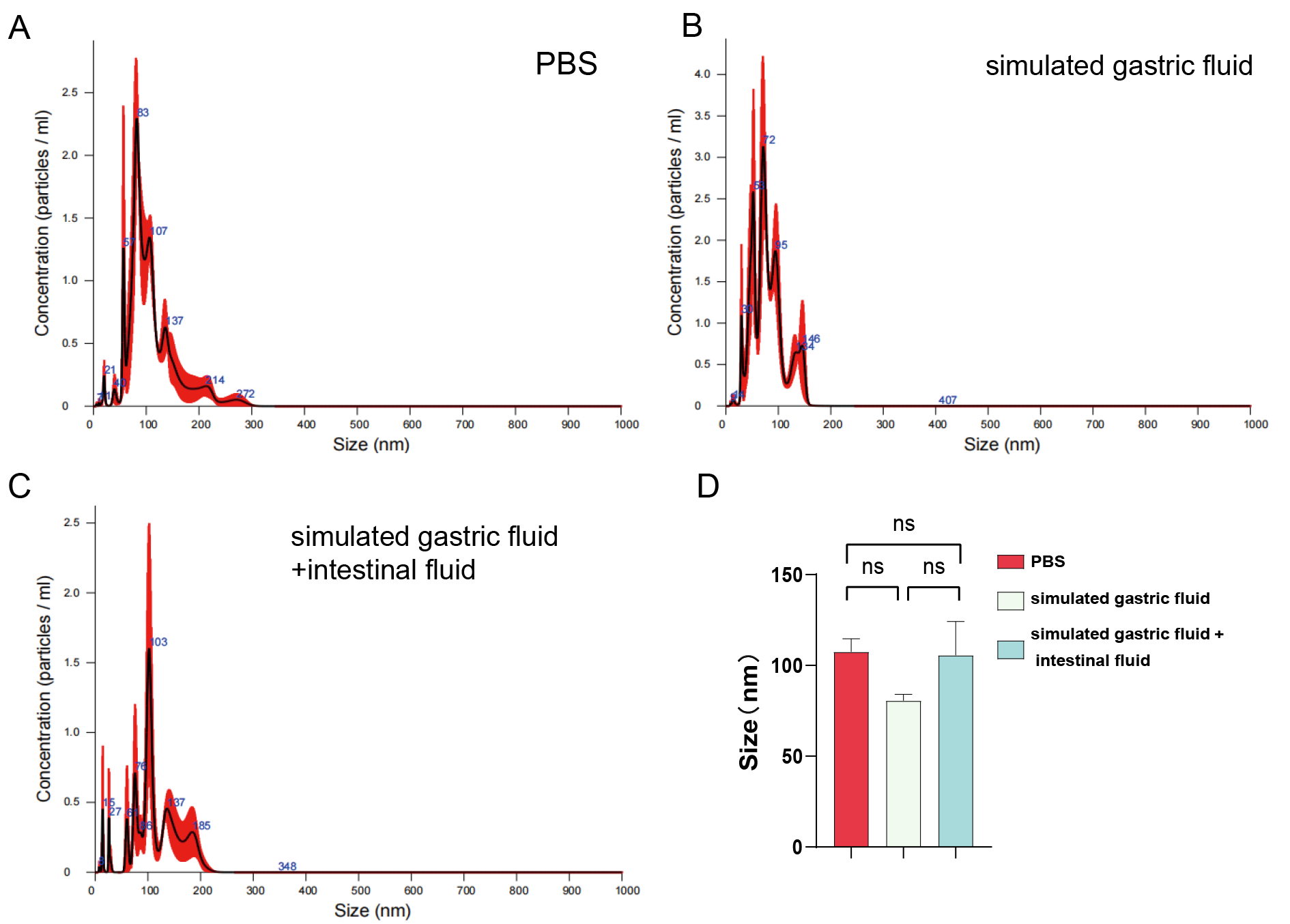
**

**Fig. S3 In vitro digestion of CDNVs.**

(A)NTA analysis of CDNVs resuspended in PBS. (B) NTA analysis of CDNVs digested in simulated gastric fluid. (C) NTA analysis of CDNVs that digested in simulated gastric fluid and intestinal fluid. (D) Statistical analyses of the average particle size of the three types of CDNVs. The results represent 3 independent experiments (n=3). Data represent means ± SD. ns, not significant.

**
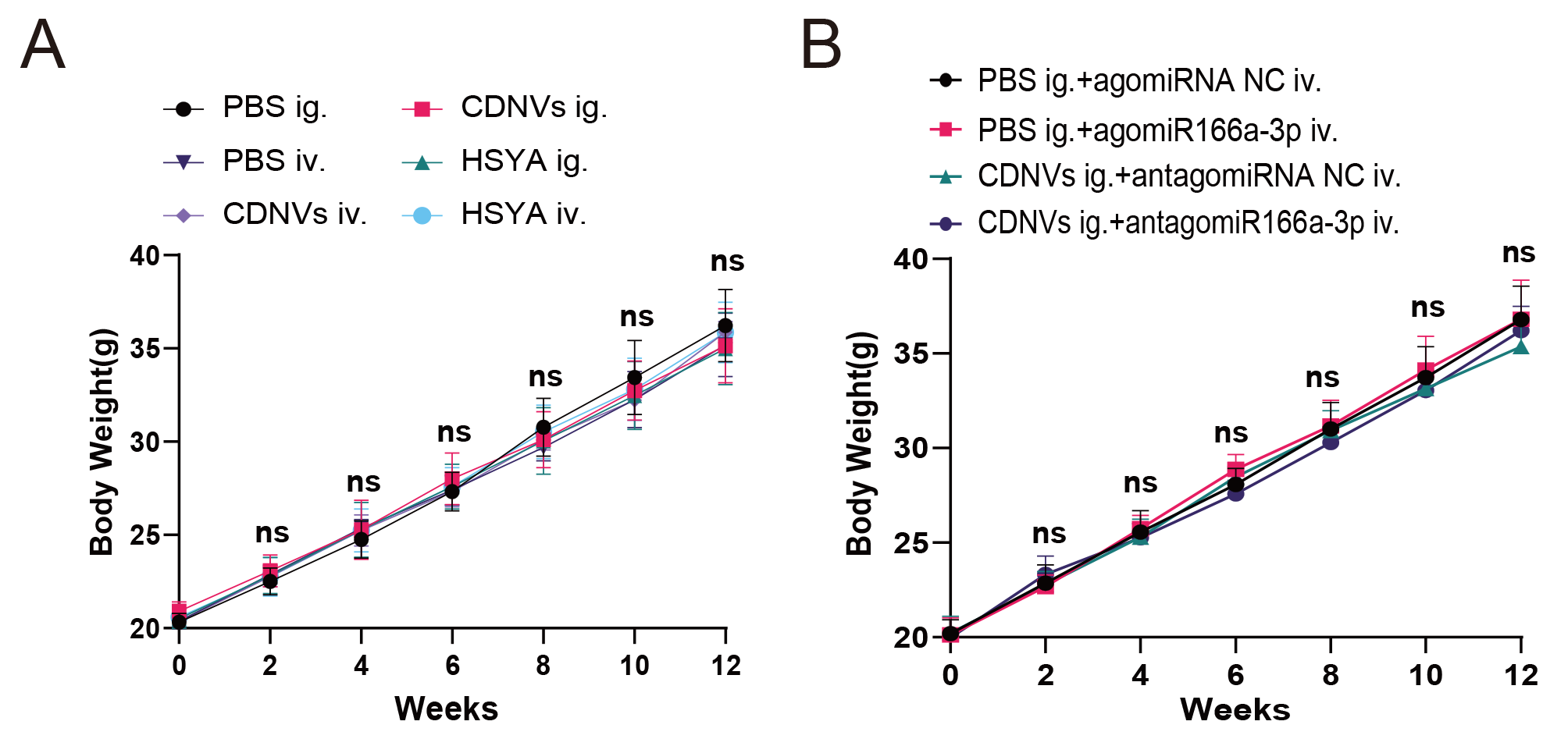
**

**Fig. S4 Body weight of ApoE-/- mice.**

(A) Statistical analyses of changes in body weight in the indicated groups (n = six mice per group). Data represent means ± SD. (B) Statistical analyses of changes in body weight in the indicated groups (n = six mice per group). Data represent means ± SD. ns, not significant.

**
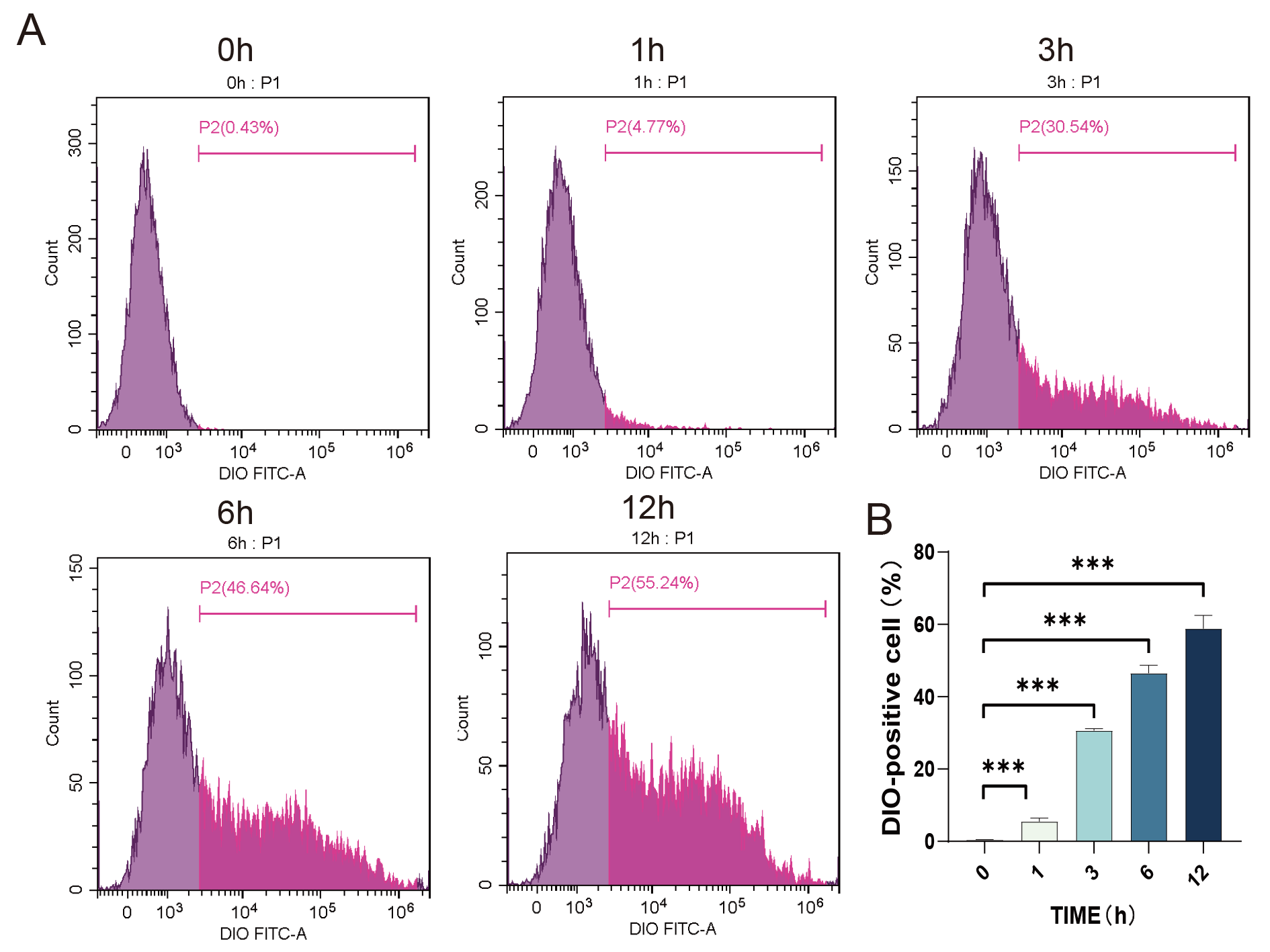
**

**Fig. S5 Uptake efficiency of CDNVs by HUVECs.**

(A) Flow cytometry-based assessment of uptake efficiency of DIO-CDNVs by HUVECs. (B) Statistical analyses of the percentage of DIO-positive cells. The results represent three independent experiments (n=3). Data represent means ± SD. ***P <0.001.


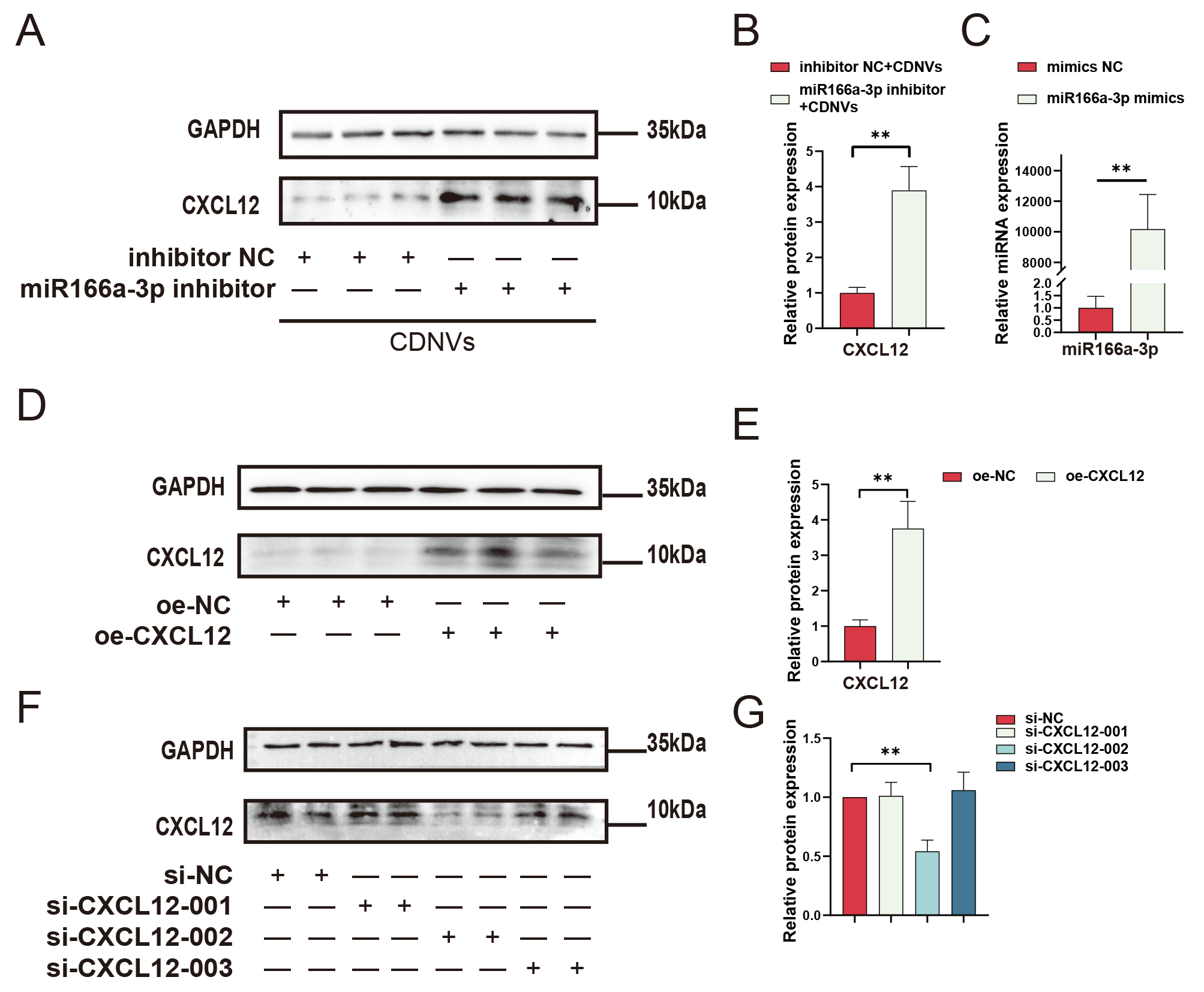


**Fig. S6 Construction of miR166a-3p mimics, miR166a-3p inhibitor, oe-CXCL12, and si-CXCL12.**

(A)Western blot assay to validate the protein level of CXCL12 in HUVECs transfected with miR166a-3p inhibitor and related statistical analysis. inhibitor NC represents the control. (B) The graphs show the quantification of CXCL12 proteins normalized to GAPDH. (C) q-PCR assay to evaluate the level of miR166a-3p in HUVECs transfected with miR166a-3p mimics. mimics NC represents the control. The graphs show the quantification of the miR166a-3p normalized to U6. (D) Western blot assay to validate the effect of CXCL12 overexpression (oe-CXCL12). oe-NC represents the control. (E) The graphs show the quantification of CXCL12 proteins normalized to GAPDH. (F) Western blot assay to validate the effect of CXCL12 knockdown (si-CXCL12). Si-NC represents the control. (G) The graphs show the quantification of CXCL12 proteins normalized to GAPDH. The results represent three independent experiments (n=3). Data represent means ± SD. **P <0.01.

**2. Supplementary table**

**Table S1 Primers for quantitative RT‒PCR analysis**

| Name | Sequence (5′→3′) |
| --- | --- |
| *hsa-GAPDH* | F: TCGGAGTCAACGGATTTGGT |
|  | R: TTCCCGTTCTCAGCCTTGAC |
| *hsa-ICAM-1* | F: GCAACCTCAGCCTCGCTAT |
|  | R: ACAACTTGGGCTGGTCACAG |
| *hsa-VCAM-1* | F: GGACCACATCTACGCTGACAA |
|  | R: CTCCAGAGGGCCACTCAAAT |
| *hsa-CXCL12* | F: ATTCTCAACACTCCAAACTGTGC |
|  | R: ACTTTAGCTTCGGGTCAATGC |
| *hsa-U6-RT* | CTCAACTGGTGTCGTGGAGTCGGCAATTCAGTTGAGAAAAATATG |
| *hsa-U6* | F: CTCGCTTCGGCAGCACA |
|  | R: AACGCTTCACGAATTTGCGT |
| *Mmu-U6-RT* | CGCTTCACGAATTTGCGTGTCAT |
| *Mmu-U6* | F: GCTTCGGCAGCACATATACTAAAAT |
|  | R: CGCTTCACGAATTTGCGTGTCAT |
| *miR159a-RT* | GTCGTATCCAGTGCGTGTCGTGGAGTCGGCAATTGCACTGGATACGACUAGAGC |
| *miR159a* | F: GCCGAGUUUGGAUUGAAGGGA |
|  | R: CAGTGCGTGTCGTGGAGT |
| *miR166a-3p-RT* | GTCGTATCCAGTGCGTGTCGTGGAGTCGGCAATTGCACTGGATACGACGGGGAA |
| *miR166a-3p* | F: GCTCGGACCAGGCTTCA |
|  | R: CAGTGCGTGTCGTGGAGT |
| *miR170-5p-RT* | GTCGTATCCAGTGCGTGTCGTGGAGTCGGCAATTGCACTGGATACGACTCTGAG |
| *miR170-5p* | F: GCCGTATTGGCCTGGTTCA |
|  | R: CAGTGCGTGTCGTGGAGT |
| *miR393-5p-RT* | GTCGTATCCAGTGCGTGTCGTGGAGTCGGCAATTGCACTGGATACGACGGATCA |
| *miR393-5p* | F: GCUCCAAAGGGAUCGCAU |
|  | R: CAGTGCGTGTCGTGGAGT |
| *miR396f-RT* | GTCGTATCCAGTGCGTGTCGTGGAGTCGGCAATTGCACTGGATACGACCAGTTC |
| *miR396f* | F: GCCGTTCCACGGCTTTCTT |
|  | R:CAGTGCGTGTCGTGGAGT |
